# Ultrabroadband Directional Tunable Thermal Emission Control Based on Vanadium Dioxide Photonic Structures

**DOI:** 10.1002/advs.202416437

**Published:** 2025-02-20

**Authors:** Qixiang Chen, Chengcong Li, Xuemei Huang, Yuehui Lu, Hua Xu, Yang An, Longnan Li, Wei Li, Xiaobo Yin, Xun Cao, Dongliang Zhao

**Affiliations:** ^1^ School of Energy and Environment Southeast University Nanjing Jiangsu 210096 P. R. China; ^2^ State Key Laboratory of High‐Performance Ceramics and Superfine Microstructure Shanghai Institute of Ceramics Chinese Academy of Sciences Shanghai 200050 P. R. China; ^3^ Center of Materials Science and Optoelectronics Engineering University of Chinese Academy of Sciences Beijing 100049 P. R. China; ^4^ Zhejiang Provincial Engineering Research Center of Energy Optoelectronic Materials and Devices Ningbo Institute of Materials Technology and Engineering Chinese Academy of Sciences Ningbo Zhejiang 315201 P. R. China; ^5^ School of Physical Science and Technology Ningbo University Ningbo Zhejiang 315211 P. R. China; ^6^ GPL Photonics Laboratory Key Laboratory of Luminescence Science and Technology Chinese Academy of Sciences & State Key Laboratory of Luminescence and Applications Changchun Institute of Optics Fine Mechanics and Physics Chinese Academy of Sciences Changchun Jilin 130033 P. R. China; ^7^ Department of Mechanical Engineering The University of Hong Kong HongKong 999077 P. R. China; ^8^ Department of Physics The University of Hong Kong Hong Kong 999077 P. R. China; ^9^ Institute of Science and Technology for Carbon Neutrality Southeast University Nanjing Jiangsu 210096 P. R. China; ^10^ Institute for Carbon Neutral Development Southeast University Nanjing Jiangsu 210096 P. R. China

**Keywords:** directional thermal emission, nanomaterial, thermal radiation, thermochromism, vanadium dioxide

## Abstract

The manipulation of thermal radiation amplitude or direction over a broadband spectrum is a fundamental capability, demonstrating significant potential in thermal management and infrared information encryption. However, existing approaches cannot control both aspects simultaneously. In this study, an ultrabroadband directional tunable thermal emitter (UDTTE) utilizing the metal‐insulator transition properties of vanadium dioxide photonic structure and the Brewster effect is proposed. Before the phase transition, the UDTTE exhibits an average emissivity as low as 0.07 across the 3–20 µm band and the entire range of angles. After the phase transition, the UDTTE maintains a low emissivity of 0.33 for incident angles below 73° but displays a high emissivity of 0.78 in the 73°–83° range. This designed experiments in information encryption demonstrate that the UDTTE can synergistically utilize temperature, viewing angle, and polarization to achieve multi‐level encryption of IR information. This strategy further enhances the capability to manipulate thermal radiation and holds promise for advancing technologies in information security, IR camouflage, and thermal management.

## Graphical abstract

This work proposes an ultrabroadband directional tunable thermal emitter (UDTTE) based on VO₂ and the Brewster effect, capable of simultaneously controlling the amplitude and direction of thermal radiation. Through information encryption demonstrations, the UDTTE synergistically utilizes temperature, viewing angle, and polarization for multi‐level thermal information encryption, promising lasting impacts on information security and thermal camouflage.

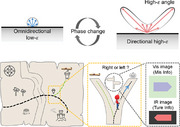

## 1 Introduction

All objects in nature spontaneously emit thermal radiation due to the oscillatory leaps of electrons and molecules, as well as lattice vibrations.^[^ ^1^, ^2^, ^3^ ^]^ In recent years, the rapid development of nanophotonics has significantly advanced spectral engineering,^[^ ^3^, ^4^, ^5^, ^6^, ^7^, ^8^, ^9^ ^]^ enabling independent control over the spectral band and magnitude of thermal emission. This progress has driven advancements in technologies such as radiative cooling and infrared (IR) camouflage.^[^ ^10^, ^11^, ^12^, ^13^, ^14^, ^15^, ^16^, ^17^, ^18^, ^19^, ^20^, ^21^, ^22^, ^23^, ^24^, ^25^, ^26^ ^]^ In contrast, directional thermal emission has developed more slowly due to the complexity of its design principles and implementation.^[^ ^4^ ^]^ Studies have indicated that the lack of directionality in thermal emission can result in reduced device efficiency.^[^ ^17^, ^27^, ^28^, ^29^ ^]^ For example, at lower ambient temperatures, thermal emission at large angles may reduce the cooling power of radiative coolers.^[^ ^29^ ^]^ Current researchers have explored various methods to achieve directional thermal emission, including surface plasmon polariton grating structures,^[^ ^4^, ^30^ ^]^ epsilon‐near‐zero (ENZ) materials,^[^ ^12^, ^27^, ^31^, ^32^, ^33^, ^34^, ^35^ ^]^ and the Brewster effect.^[^ ^17^, ^36^, ^37^ ^]^ For instance, a 1D grating structure on a silicon carbide substrate has been demonstrated to achieve directional thermal emission at a single wavelength of 11.36 µm (p‐polarization), but its manufacturing process is complex and the operational bandwidth is very narrow.^[^ ^38^ ^]^ The concept of gradient ENZ has been introduced to address the trade‐off between directionality and narrow bandwidth in directional thermal emission.^[^ ^27^ ^]^ By superimposing Berreman modes of different ENZ materials along the depth direction, the operational bandwidth for directional thermal emission can be extended to a broader range (7.7–11.5 µm or 10.0–14.3 µm, p‐polarization). However, extending the bandwidth through multi‐layer stacking introduces additional unwanted omnidirectional modes and increases the complexity of fabrication. Moreover, the limited availability of ENZ materials in nature poses a significant challenge to achieving ultrabroadband directional thermal emission using the gradient ENZ approach.

To date, the practical uses of polarization‐dependent directional thermal emission remain unclear. Utilizing the Brewster effect to achieve polarization‐dependent directional thermal emission in the 3–5 µm and 8–14 µm bands shows potential for information encryption applications. However, its encryption effectiveness is significantly limited, as encrypted patterns can be easily recognized by the human eyes.^[^ ^17^ ^]^ Combining directional thermal emission with visible/IR dual‐band camouflage may offer a promising pathway to enhance its practicality.^[^ ^14^, ^39^, ^40^ ^]^ For example, Franklin et al. developed a cavity structure that induces plasmonic resonance. By varying the cavity dimensions, it is possible to independently manipulate IR emissivity without altering the visible color, thereby achieving patterns visible only under IR detectors.^[^ ^14^ ^]^ Nonetheless, integrating these two approaches remains a significant challenge. Furthermore, although independently controlling the magnitude and direction of thermal radiation has shown potential in radiative cooling and information encryption, respectively, no known method currently allows simultaneous control of the magnitude of directional thermal emission. Confining broadband thermal radiation to a fixed direction while controlling its magnitude would fundamentally enhance the ability to manage thermal radiation, potentially advancing the fields of radiative cooling and advanced information security.

In this work, we designed and demonstrated a strategy to achieve amplitude‐tunable directional thermal emission over an ultrabroadband range of 3–20 µm by leveraging the metal‐insulator transition (MIT) properties of vanadium dioxide (VO_2_) and the pseudo‐Brewster effect. The proposed ultrabroadband directional tunable thermal emitter (UDTTE) exhibits a low emissivity of 0.07 across the 3–20 µm range and all angles before the MIT occurs. After the MIT, the emissivity remains low at 0.33 for angles below 73° but increases to 0.78 within the 73°–83° angle range. Furthermore, we experimentally demonstrated the application potential of UDTTE in information encryption. We showcased the use of UDTTE for attacker‐misleading information encryption, where the true information carried by UDTTE can only be decrypted under conditions of high temperature (>68 °C), large viewing angles (73°–83°), and p‐polarization state of the polarizer. Additionally, the UDTTE features a simple structure, is easily scalable for manufacturing, and is compatible with both rigid and flexible substrates, demonstrating significant potential for practical applications.

## 2 Results and Discussion

**Figure 1:**
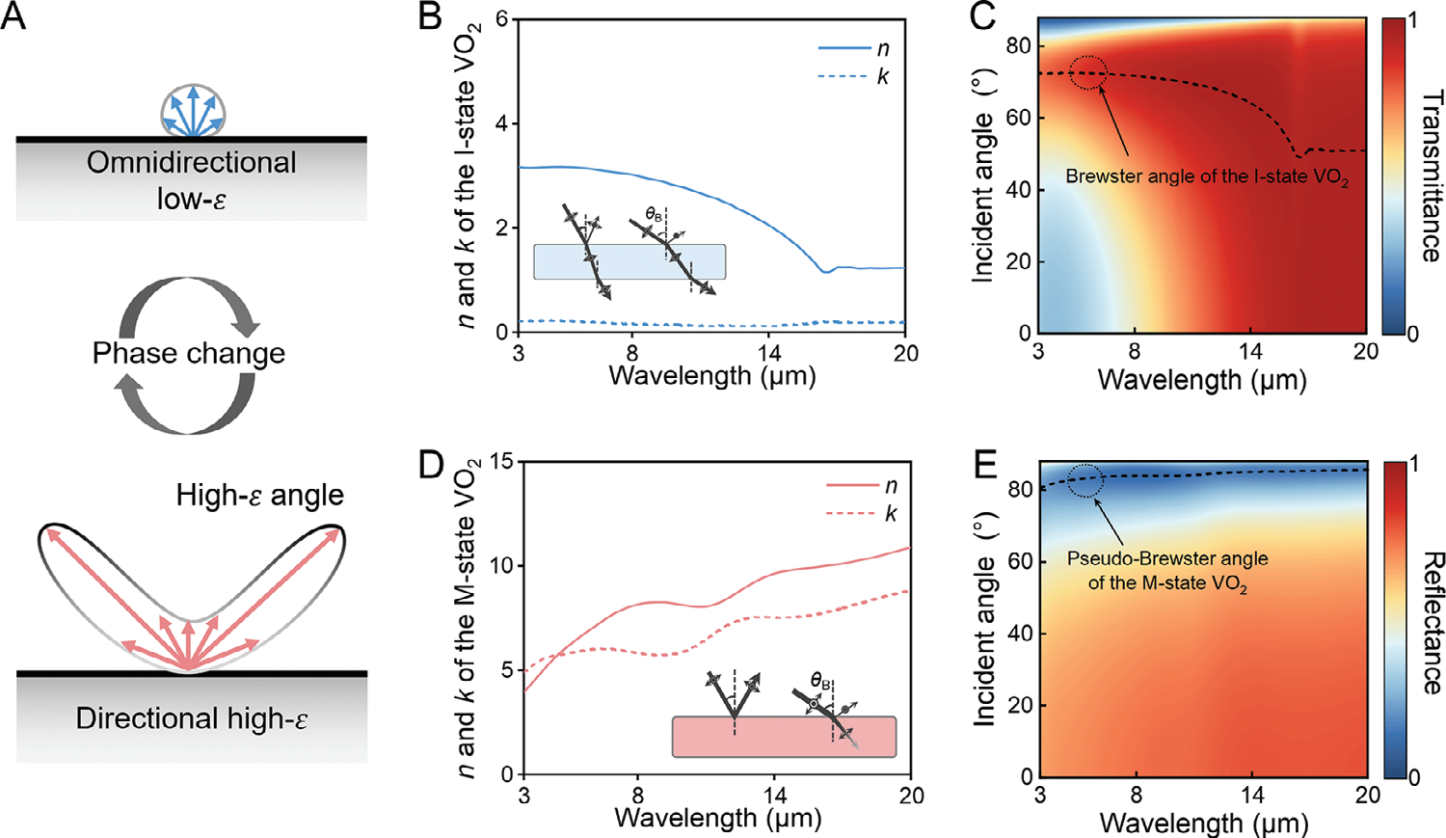
Principle of ultrabroadband directional tunable thermal emission (UDTTE). A) Schematic of an omnidirectional low‐𝜀 and a directional high‐𝜀 object. B) Complex refractive index of the I‐state VO_2_, with the inset showing the propagation of thermal emission at different incidence angles upon the M‐state VO_2_. C) Calculated angle‐dependent transmittance spectra of a 300‐nm‐thick I‐state VO_2_ film, with dashed lines indicating the calculated Brewster angle. D) Complex refractive index of the M‐state VO_2_, with the inset showing the propagation of thermal emission at different incidence angles upon the M‐state VO_2_. E) Calculated angle‐dependent reflectance spectra of a 300‐nm‐thick M‐state VO_2_ film, with dashed lines indicating the calculated pseudo‐Brewster angle.

The concept of the proposed UDTTE is illustrated in **Figure** **1**A, which demonstrates that the UDTTE can switch between states of directional high‐emission (𝜀) and omnidirectional low‐𝜀. This mechanism is based on the significant differences in the refractive index (*n*) and extinction coefficient (*k*) resulting from the MIT of VO_2_. As shown in Figure 1B, when the temperature is below the MIT temperature (≈68 °C), VO_2_ is in the insulating state (I‐state). In this state, VO_2_ has a relatively low refractive index and an extinction coefficient close to 0 in the mid‐infrared (MIR) range.^[^ ^41^ ^]^ Therefore, I‐state VO_2_ incurs almost no loss (i.e., low absorption/reflection) for electromagnetic waves incident at any angle within this wavelength range. The calculated angle‐dependent transmittance spectra (Figure 1C and p‐polarization) indicate that I‐state VO_2_ is highly transparent to electromagnetic waves in the 8–20 µm range. In the 3–8 µm range, a noticeable enhancement in transmittance due to the Brewster effect can be observed (θ_B (i − state)_ = tan ^−1^(*n*), where *n* represent the refractive index of the I‐state VO_2_).^[^ ^17^, ^42^ ^]^

However, when the temperature exceeds 68 °C, VO_2_ transitions to the metallic state (M‐state), significantly increasing its refractive index and extinction coefficient in the MIR range (Figure 1D). This results in high reflectivity similar to that of metals.^[^ ^43^, ^44^, ^45^ ^]^ At the pseudo‐Brewster angle for the M‐state VO_2_, the reflection is minimized (i.e., absorption/emission is maximized): $\theta_{B\;(M-\mathrm{state})}=\cos^{-1}\left(\frac{1}{\sqrt{n^{2}+k^{2}}}\;\right)$, where *n* and *k* represent the refractive index and extinction coefficient of the M‐state VO_2_, respectively (Note S1, Supporting Information).^[^ ^46^ ^]^ The angle‐dependent reflectance spectra (p‐polarization) of M‐state VO_2_ shown in Figure 1E demonstrate the specific directional decrease in reflectance due to the pseudo‐Brewster effect, indicating enhanced directional absorption/emission (p‐polarization).

**Figure 2:**
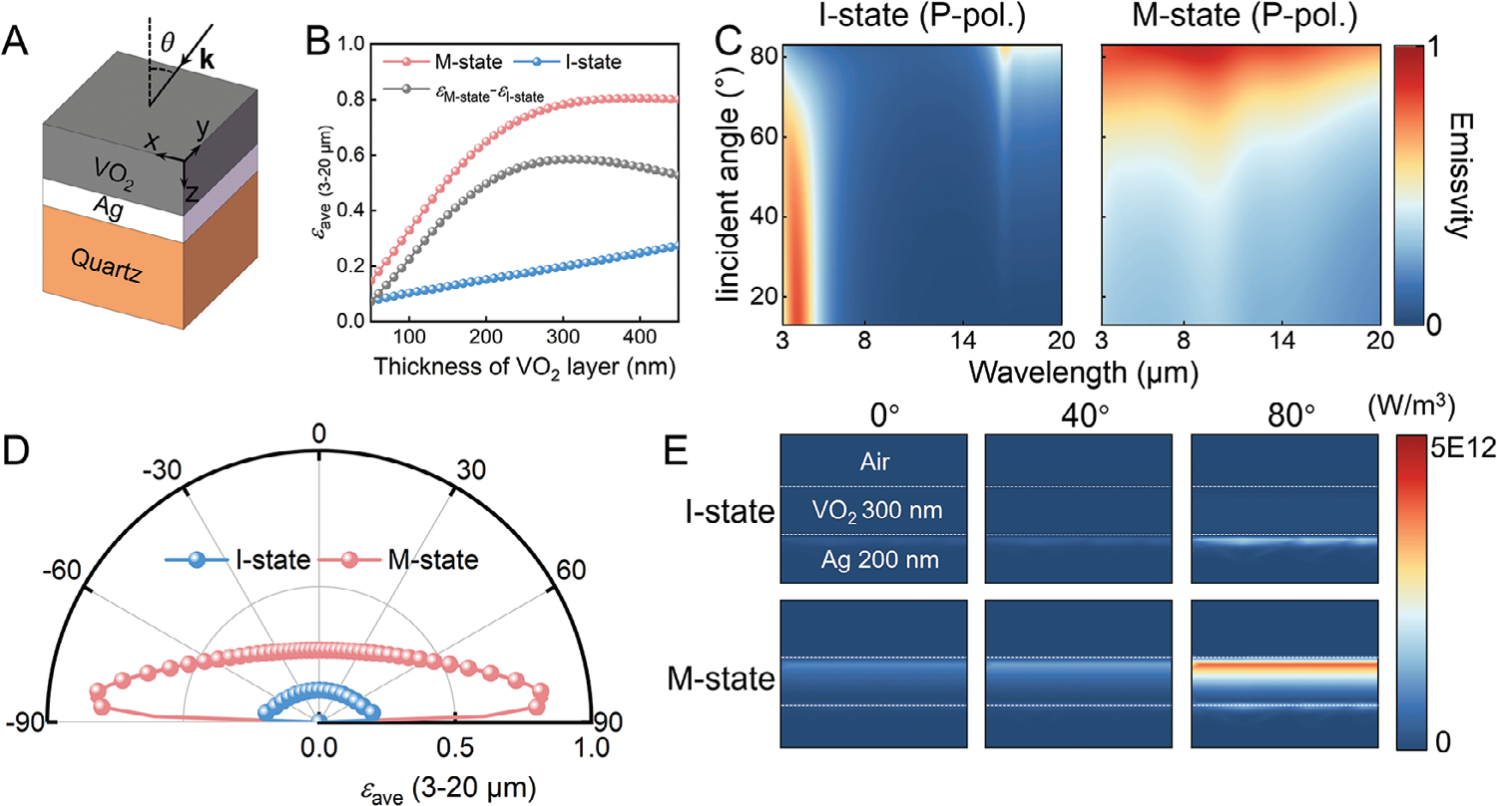
Calculated emissivity spectral and power loss density distribution (p‐polarization) of the UDTTE. A) Schematic diagram of the UDTTE. B) The relationship between the thickness of the VO₂ layer and the average emissivity for the VO₂/Ag (200 nm)/quartz (1mm) structure at an incidence angle of 84°. C) Calculated angle‐dependent emissivity spectra of the UDTTE before (left) and after (right) the MIT. D) The average emissivity of the UDTTE over the 3–20 µm range. E) Calculated power loss density distribution of the UDTTE at a wavelength of 20 µm.

The directional thermal emission of the UDTTE originates from the low reflectance of M‐state VO_2_ at the pseudo‐Brewster angle, while the control of the directional thermal emission magnitude is due to the high transmittance of I‐state VO_2_ for electromagnetic waves over the entire angular range. This requires that the film layer or substrate beneath the VO_2_ film exhibit a sufficiently low loss in the MIR range, meaning it should maintain high reflectance or transmittance at any incidence angle within the MIR range. Metal Ag is well‐known for its high reflectance in the MIR range. Although there is a slight decrease in reflectivity at the pseudo‐Brewster angle (≈88°) (Figure S1, Supporting Information), Ag can still be considered a highly reflective layer at any incident angle within the MIR range. As shown in **Figure** **2**A, we used the finite element method to calculate the emissivity spectral (p‐polarization) and power loss density distribution of a UDTTE (VO_2_ /200‐nm‐thick Ag/1‐mm‐thick Quartz) over the 3–20 µm range, the thickness of the VO₂ layer is determined by the average emissivity difference of the UDTTE at an incidence angle of 84° before and after the MIT (Figure 2B). This difference reaches its maximum when the VO₂ thickness is ≈300 nm. Therefore, the optimal thickness of VO₂ is determined to be 300 nm. The material refractive indices and calculated results by the transmission matrix method are shown in Figures S2 and S3 (Supporting Information).^[^ ^41^, ^47^, ^48^ ^]^ Before the MIT, the average emissivity over all incident angles in the 3–20 µm range was as low as 0.15 (left panel of Figure 2C and blue dashed line in Figure 2D). After MIT, the average emissivity remains low at 0.35 for incident angles less than 68°. However, due to the pseudo‐Brewster effect, it increases to 0.76 for larger incidence angles in the range of 74°–86°, reaching a maximum value of 0.84 at 84° (right panel of Figure 2C, red dashed line in Figures 2D; Figure S4, Supporting Information). The calculated emissivity spectra for s‐polarization and unpolarized are also shown in Figure S5 (Supporting Information). To demonstrate the energy dissipation of the UDTTE for incident waves, we calculated the power loss distribution of p‐polarization electromagnetic waves with a wavelength of 20 µm incident on the UDTTE at different angles. The results shown in Figure 2E indicate that before the MIT, the transparent VO₂ layer allows electromagnetic waves to pass through and be reflected by the underlying Ag layer (Figure 1C), resulting in minimal dissipation for waves at any incident angle. However, after the MIT, as the incident angle increases to 80°, the pseudo‐Brewster effect causes the reflection of p‐polarized light to vanish (Figure 1E). The waves entering the VO₂ layer are dissipated due to its high extinction coefficient (Figure 1D), resulting in high absorption/emissivity. Furthermore, the emissivity spectra and power loss distributions of VO₂ after deposition on IR‐transparent Barium fluoride (BaF₂) and IR‐absorbing quartz substrates were also calculated (Figures S6–S8, Supporting Information). Compared to other IR‐transparent substrates, BaF₂ exhibits higher IR transmittance in the 3–14 µm range and demonstrates relatively greater stability in the air, making it the preferred choice for the IR‐transparent substrate). Our calculations indicate that the VO_2_ films on a BaF_2_ substrate can modulate the magnitude of directional thermal emission in the 3–14 µm range. In contrast, the intrinsic high absorption of a quartz substrate results in a high emissivity of the VO_2_ film even before the phase transition, preventing effective control of directional thermal emission. Table S1 (Supporting Information) provides a detailed comparison of VO₂ performance on three different substrates in terms of directional thermal emission, tunability, control mechanism, operating spectral range, and fabrication costs.

**Figure 3:**
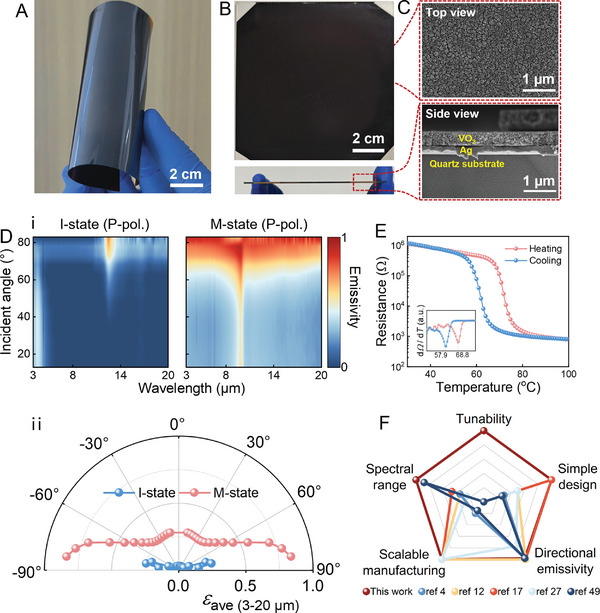
Fabrication and characterization of the UDTTE. Optical photographs of flexible (A) and rigid (B) UDTTE. (C) SEM photographs of rigid UDTTE. D) Measured angle‐dependent emissivity spectra (p‐polarization) of the UDTTE in the 3–20 µm range (i) and averaged emissivity of the UDTTE in the 3–20 µm range as a function of angle before and after the MIT (ii). E) Measured resistance of the UDTTE during the heating and cooling processes. F) Performance comparison of the UDTTE with other broadband directional thermal emitters.

The above‐calculated results indicate that fabricating VO_2_ on an Ag layer can simultaneously meet the requirements for ultrabroadband (3–20 µm), directional, and tunable thermal emission. In the actual fabrication process, Ag (200 nm)/VO_2_ (300 nm) layers were deposited on the flexible‐substrate polyimide (10 cm × 10 cm/100‐µm‐thick) and the rigid‐substrate quartz (10 cm × 10 cm/1‐mm‐thick), respectively (Figure S9, Supporting Information). The resulting optical photographs of the fabricated UDTTEs are shown in **Figure** **3**A, B. The cross‐sectional photographs captured by scanning electron microscope (SEM) demonstrate the layered structure of the UDTTE (Figure 3C). The actual thicknesses of the Ag and VO_2_ layers are 305 and 190 nm, respectively, which are very close to the theoretical design thicknesses. The surface‐scanned energy dispersive spectroscopy spectrum also clearly shows the elemental distribution of the UDTTE's layered structure (Figure S10, Supporting Information).

To accurately assess the performance of the UDTTE's directional and tunable thermal emission characteristics, we measured the angle‐dependent emissivity spectra (p‐polarization) of the UDTTE at low (26 °C) and high (90 °C) temperatures. As shown in Figure 3D (i) (left) and Figure 3D (ii) (blue dashed line), the UDTTE maintains low emissivity across all angles and wavelengths before MIT. After the MIT, it exhibits significant directional thermal emission characteristics. Specifically, before MIT, the average emissivity across all angles is as low as 0.07. After MIT, the average emissivity remains low at 0.33 for angles below 73° but increases to 0.78 for angles between 73° and 83° (as shown in Figure 3D (i) (right) and the red dashed line in Figure 3D (ii)). The measured emissivity spectra under s‐polarization and unpolarized confirm that directional thermal radiation occurs only under p‐polarization (Figure S11, Supporting Information). The temperature‐dependent resistance test (Figure 3E) indicates that the prepared VO_2_ film exhibits temperature‐dependent MIT behavior, with a resistance change of up to three orders of magnitude before and after the MIT, demonstrating the high quality of the VO_2_ film. In addition, the prepared VO₂ film exhibits no significant changes in phase transition performance after 1000 high‐low temperature cycling tests, indicating excellent durability and stability (Figure S12, Supporting Information). Compared to other directional thermal emitters (Figure 3F),^[^ ^4^, ^12^, ^17^, ^27^, ^49^ ^]^ the UDTTE not only features tunability, an ultrabroadband operating bandwidth, and a simple structure but also exhibits comparable or superior performance in terms of directional emissivity and scalable manufacturing, making it a highly attractive thermal emitter. Additionally, we fabricated UDTTEs on both IR‐transparent BaF_2_ and IR‐absorbing quartz substrates and then measured their emissivity spectra, as detailed in Figures S13 and S14 (Supporting Information). The results confirmed that the BaF_2_ substrate is also suitable for fabricating UDTTEs, though their operational bandwidth is limited to the 3–14 µm range due to the IR transmittance of BaF_2_. Although the bandwidth is limited, BaF₂ is also transparent to visible light, making BaF₂‐based UDTTE promising for applications in transparent materials such as windows, thereby expanding its application scope. In addition, although quartz substrates enable directional tunable thermal emission; however, the inherent high absorption of quartz restricts its tuning mechanism to states between omnidirectional high emissivity and directional high emissivity, which does not align with the objectives of applications such as thermal camouflage or information encryption.

**Figure 4:**
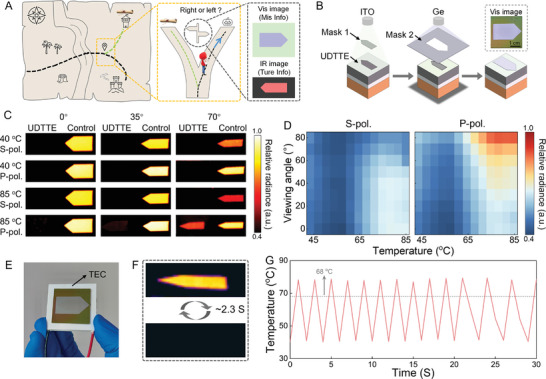
Experimental demonstration of UDTTE implementing multi‐level IR information encryption. A) A schematic of the information‐misdirection scenario using the UDTTE, along with images of the misdirection arrow patterns captured under visible and an IR camera (the IR photographs are assumed to be taken at temperatures above 68 °C, under p‐polarization, and at 70° viewing angle). B) Schematic of the experimental flow for achieving the information misdirection function based on UDTTE. C) IR photographs of the UDTTE with camouflaged arrow patterns (left region of the subfigure) and a high emissivity control sample of the arrow patterns (right region of the subfigure) at varying temperatures, viewing angles, and polarization. D) Relative radiance of the UDTTE at different temperatures, polarizations, and viewing angles. E) Optical image of the UDTTE integrated with the TEC. F) IR images of the TEC‐based fast temperature‐switching device in the decryption (top) and encryption (bottom) states. G) Temperature profile of the fast temperature‐switching device under cyclic forward/reverse voltage application.

One of the hallmarks of UDTTE is the capability to emit a substantial amount of heat at specific temperate, angle, and polarization conditions. To further explore the application potential of the UDTTE, we conducted a misinformation encryption demonstration, as illustrated in **Figure** **4**A. In this demonstration, the UDTTE transmits a right‐turned arrow as misleading information in visible light. However, the true information is encrypted at multiple levels using the UDTTE's directional tunable thermal radiation properties. The true directional information becomes visible only in an IR detector under specific conditions: high temperature (>68 °C), p‐polarization, and a specific viewing angle range (73°–83°). The UDTTE used for this misinformation demonstration was fabricated according to the process outlined in Figure 4B. The left‐pointing arrow was created by depositing a 250 nm Indium Tin Oxide (ITO) layer onto the UDTTE through an arrow‐shaped mask, while the right‐pointing arrow was formed by depositing a 50 nm Ge layer using an opposite arrow‐shaped mask. The ITO layer serves as the low‐temperature background environment for IR imaging, while its thickness‐sensitive visible color is used to display visible color patterns (the variation in visible color of the ITO layer in Figure 4B is caused by thickness non‐uniformity, Figure S15, Supporting Information). The Ge layer, with its IR transparency and visible opacity, is employed to conceal IR patterns from the human eyes while maintaining the thermal emission properties of the UDTTE (Figure S16, Supporting Information). Additionally, the stability of ITO and Ge in air protects the underlying VO₂ layer from oxidation, as evidenced by the unchanged IR apparent temperature of the fabricated encryption device after 4 months of exposure to air, demonstrating excellent durability of its optical properties (Figure S17, Supporting Information). It should be noted that other metals, such as Al or Ag, can replace ITO as the low‐temperature background layer. These metals exhibit high reflectivity even at thin thicknesses. Although their color contrast with Ge is lower compared to ITO, their thinner thickness minimizes the thickness difference between the metal layer and Ge, reducing the visibility of pattern edges under certain angles to the human eyes (Figure S18, Supporting Information). Figure 4C displays IR photographs of the UDTTE with camouflaged arrow patterns (left region of the subfigure) and a high emissivity control sample (0.93) of the arrow patterns (right region of the subfigure) under different temperatures, angles, and polarizations (the schematic of the measurement setup and the IR photographs comparing the UDTTE without camouflage patterns to the control sample are shown in Figure S19, Supporting Information). The high emissivity characteristics of the contrast sample (right region of the subfigure), which are independent of temperature, viewing angle, and polarization, result in high relative radiance under any condition (Notes S2, S3, Supporting Information), making its thermal radiation signal easily detectable by IR detectors. In contrast, the UDTTE (left region of the subfigure) exhibits significant temperature, angle, and polarization‐dependent selective thermal emission characteristics, showing high relative radiance only under high temperature, large viewing angle, and p‐polarization conditions. The thermal maps in Figure 4D display the relative radiance distribution of the UDTTE as a function of temperature (45–85 °C), angle (0°–80°), and polarization, indicating the difficulty of decoding the IR information carried by the UDTTE (Figure S20, Supporting Information also show the relative radiance distribution of the control sample and quartz‐based UDTTE). The information encryption method based on temperature, angle, and polarization implies that decryption relies not only on the polarization state and viewing angle of the detector but also requires the decryptor to actively apply temperature to transition the UDTTE to an observable state for decryption. Furthermore, to enhance the practicality of the UDTTE, we integrated it with a thermoelectric cooler (TEC) to fabricate a compact, fast‐response cyclic switching device (Figure 4E). By applying forward/reverse voltages, the device can achieve high‐low temperature switching between 40 and 80 °C within 2.3 s (Figure 4F), a temperature range that fully spans the phase transition of VO₂. The device demonstrates excellent continuous switching stability, completing 13 switching cycles within 30 s (Figure 4G). In addition, in previous work,^[^ ^50^ ^]^ we have verified that the MIT temperature of VO₂ can be flexibly tuned by doping with different proportions of tungsten element. It is expected that this method can further enhance the response speed of the device and improve its practicality. The proof‐of‐concept experiments mentioned above demonstrate the tremendous potential of UDTTE in the field of information security, promising significant advancements in this domain.

## 3 Conclusion

In summary, we propose a UDTTE based on VO_2_ and the Brewster effect, characterized by a high emissivity of 0.78 that occurs exclusively within the M‐state, p‐polarization, and a specific angle range (73°–83°). At incident angles below 73°, the average emissivity is as low as 0.33, while in the I‐state, it maintains a low emissivity of 0.07 across the entire angle range. Our experiments demonstrate that UDTTE can achieve multi‐level information encryption through both active (temperature‐dependent) and passive (angle and polarization‐dependent) methods. This research significantly enhances the ability to manipulate thermal radiation, providing unique insights for more complex spectral engineering and demonstrating the application potential of polarization‐dependent directional thermal radiation. Additionally, the UDTTE exhibits a simple structure, enabling low‐cost and large‐scale manufacturing, which holds promise for advancing the development of advanced information security, and thermal management applications.

## 4 Experimental Section

### Numerical Simulations

Using COMSOL Multiphysics software simulated the emissivity spectra and power loss distribution of the UDTTE under p‐polarization with simplified in‐plane 2D periodic boundary conditions. The emissivity spectra under s‐polarization were simulated using out‐of‐plane 2D periodic boundary conditions. The refractive indices of VO_2_, Ag, BaF_2_, quartz, ITO, and Ge are illustrated in Figures S2 and S15 (Supporting Information).

### Fabrication of the UDTTE

The 1‐mm‐thick quartz substrate was purchased from Beijing Shengyakang Technology Co., Ltd., and the 1 mm thick single‐crystal BaF_2_ substrate was purchased from Shanghai Puruide Optical Materials Co., Ltd. Before preparing the UDTTE, the substrates were washed in ethanol, acetone and deionized water, then blown and dried with a high‐pressure nitrogen gun. All the layers were deposited using the magnetron sputtering method. First, the Ag layer was prepared with an argon flow rate of 60 sccm using a 50 W DC power supply, and the deposition chamber pressure was maintained at 0.3 Pa. Next, the VO_2_ layer was deposited by DC sputtering using a V_2_O_3_ target (purity 99.9%) in a mixed atmosphere of argon and oxygen (Ar/O_2_ = 40:10) at room temperature, with a sputtering power of 200 W. Finally, the samples were post‐annealed in the chamber at 375 °C (flexible polyimide substrate) or 450 °C (rigid quartz substrate) under a vacuum of 5 Torr for 5 mins and then allowed to cool down naturally. Furthermore, ITO and Ge layers were deposited on the UDTTE using magnetron sputtering to demonstrate the information‐misleading scenario. The ITO layer was prepared using a 90 W DC power supply with an argon gas flow rate of 80 sccm, maintaining a deposition chamber pressure of 0.3 Pa. The Ge layer was prepared using a 60 W RF power supply with an argon gas flow rate of 45 sccm, maintaining a deposition chamber pressure of 0.4 Pa.

### Optical and Electrical Properties Characterization

The cross‐sectional SEM photographs of the UDTTE were taken using a field emission SEM (Regulus8100, Hitachi). The elemental distribution was analyzed using energy‐dispersive X‐ray spectroscopy (EDS, Ultim Max 65, Oxford). Considering that the transmittance (*T*) of the quartz substrate and Ag used in the sample preparation is ≈ ∼0 in the 3–20 µm range (Figure S21, Supporting Information), the absorptance (*A*) (and hence the emissivity, *ε* = *A* = 1−*R*) was calculated from the reflectance (*R*). The reflectance of the samples was measured at intervals of 5° between 13° and 83° using a Fourier transform IR (FTIR) spectrometer (VERTEX 70v, Bruker) equipped with a variable angle accessory (A513, Bruker). The average emissivity at each angle was calculated by weighting the spectral irradiance of the blackbody:

(1) $$\varepsilon_{ave\;\left(3-20\;\mu m\right)}=\frac{\int_{3}^{20}\varepsilon \left(\lambda ,\;\theta \right)I_{BB}\left(T,\;\lambda \right)d\lambda }{\int_{3}^{20}I_{BB}\left(T,\;\lambda \right)d\lambda }\;$$

where $I_{BB}\left(T,\;\lambda \right)\;=\frac{2hc^{2}}{\lambda^{5}}\;\frac{1}{e^{hc/\left(\lambda k_{B}T\right)}-1}$, denotes the spectral irradiance of a blackbody at a temperature of *T*. *T* is set to 299.15 and 363.15 K for calculating the average emissivity of the sample at temperatures of 26 and 90 °C, respectively. Here, λ, *h*, *k_B_*, and *c* represent wavelengths, the Planck constant, the Boltzmann constant, and the speed of light in a vacuum, respectively. Since the emissivity of BaF₂‐based UDTTE must account for both its IR reflectance and transmittance to determine emissivity using the relation *ε* = *A* = 1−*R*, the emissivity spectrum of UDTTE on a BaF₂ substrate was tested using the blackbody comparison method. For more details, please refer to Note S4 and Figure S22 (Supporting Information). The thermal hysteresis loop of the sample was obtained using a cryostat (Janis VPF‐100, Quantum Design). During the test, the cryostat was evacuated to minimize the influence of the surrounding environment on temperature. The temperature during the test was controlled and recorded using a temperature controller (Cryo‐con 32), and the resistance was recorded using a source meter (Keithley 2400).

### Multi‐Level Message Encryption Experiment

An experiment to measure the radiance at different temperatures, viewing angles, and polarizations was designed using a long‐wave IR camera (FLIR E85) with an operating wavelength range of 7.5–14 µm. During the tests, the IR camera was fixed at a distance of 50 cm from the center of the rotating stage. The UDTTE and the control sample were fixed on a vertically placed heater, with low‐emissivity Al foil covering the surface and sides of the heater to minimize the influence of heat radiation from the heater on the measurement results. A quartz substrate coated with a high‐emissivity paint (THI‐1B, Taseto, ${\bar{\varepsilon }}_{7.5-14\;\mu m}=0.93$) was used as the reference sample (Figure S21, Supporting Information). The thickness of the quartz substrate was the same as that used for the UDTTE (1 mm) to ensure the actual temperature of the UDTTE and the control sample surfaces were identical. The actual temperature of the sample was detected using a K‐type thermocouple attached to the surface and recorded by a data logger (RDXL4SD, Omega Engineering) with an uncertainty of 0.1 °C. A comparison between the heater's set temperature and the actual temperature measured by the thermocouple is shown in Figure S21 (Supporting Information). A polarizer was placed in front of the IR camera lens to obtain IR photographs under S and P polarization. A reflective board with a central hole the same size as the polarizer and covered with Al foil was placed in front of the polarizer to prevent body heat radiation from being reflected by the Al foil on the heater surface to the IR camera. The indoor temperature was controlled at 26 °C by an air conditioner during the tests. The TEC (TEC1‐12706) used in the fast temperature‐switching device operates with an applied voltage of 12 V during both heating and cooling processes.

## Conflict of Interest

The authors declare no conflict of interest.

## Author Contributions

Q.C., C.L., and X.H. contributed equally to this work. Q.C., X.H., D.Z performed conceptualization. Q.C., C.L., X.H., H.X., Y.L., Y.A., L.L., W.L., X.C., D.Z did methodology. Q.C., C.L., X.H. did investigation. Q.C., C.L., X.H., X.C., D.Z. perdormed visualization. W.L., D.Z did fund acquisition. D.Z performed project administration. X.Y., X.C., D.Z. did supervision. Q.C., X.H. wrote the original draft. Q.C., C.L., X.H., W.L., X.Y., X.C., D.Z. reviewed and did editing.

## Supporting information



Supporting Information

## Data Availability

The data that support the findings of this study are available from the corresponding author upon reasonable request.
