# Supplementary material for: Ultrabroadband Directional Tunable Thermal Emission Control Based on Vanadium Dioxide Photonic Structures
Source: Adv Sci (Weinh). 2025 Feb 20;12(15):2416437. doi: 10.1002/advs.202416437 (PMC12005815; doi:10.1002/advs.202416437)
Supplement: Supplementary file 1 — Supporting Information [file ADVS-12-2416437-s001.docx]

Supplementary Materials for

**Ultrabroadband Directional Tunable Thermal Emission Control Based on Vanadium Dioxide Photonic Structures**

*Authors, and Corresponding Authors*****:*** *Qixiang Chen ^†^, Chengcong Li ^†^, Xuemei Huang ^†^, Yuehui Lu, Hua Xu, Yang An, Longnan Li, Wei Li,* *Xiaobo Yin ^*^, Xun Cao ^*^, Dongliang Zhao ^*^*

Q. Chen, D. Zhao

School of Energy and Environment, Southeast University, Nanjing, Jiangsu 210096, China

E-mail: dongliang_zhao@seu.edu.cn

C. Li, X. Cao

State Key Laboratory of High-Performance Ceramics and Superfine Microstructure, Shanghai Institute of Ceramics, Chinese Academy of Sciences, Shanghai 200050,

China

Center of Materials Science and Optoelectronics Engineering, University of Chinese Academy of Sciences, Beijing 100049, China

E-mail: cxun@mail.sic.ac.cn

X. Huang

Zhejiang Provincial Engineering Research Center of Energy Optoelectronic Materials and Devices, Ningbo Institute of Materials Technology and Engineering, Chinese Academy of Sciences, Ningbo, Zhejiang 315201, China

Y. Lu, H. Xu

School of Physical Science and Technology, Ningbo University, Ningbo, Zhejiang 315211, China

Y. An, L. Li, W Li

GPL Photonics Laboratory, Key Laboratory of Luminescence Science and Technology, Chinese Academy of Sciences & State Key Laboratory of Luminescence and Applications, Changchun Institute of Optics, Fine Mechanics and Physics, Chinese Academy of Sciences, Changchun, Jilin 130033, P. R. China

X. Yin

Department of Mechanical Engineering, The University of Hong Kong, HongKong 999077, China

Department of Physics, The University of Hong Kong, Hong Kong 999077, China

E-mail: xbyin@hku.hk

D. Zhao

Institute of Science and Technology for Carbon Neutrality, Southeast University, Nanjing, Jiangsu 210096, China

Institute for Carbon Neutral Development, Southeast University, Nanjing, Jiangsu, 210096, China

^†^These authors contributed equally to this work.

**The file includes:**

Supplementary Texts

Figures S1 to S22

Table S1

References

Supplementary Text

Note S1. Theoretical mode for Brewster angle of metals.

For metals in an air medium, the reflectance of P-polarized electromagnetic waves is given by the following equation:^[1]^

$R\left( \theta\right)=\left| \frac{\epsilon\cos\theta-\sqrt{\epsilon-{sin}^{2}\theta}}{\epsilon\cos\theta+\sqrt{\epsilon-{sin}^{2}\theta}} \right|^{2}$ (S1)

Where $\theta$ denotes the angle of incidence. For simplicity, the surrounding environment is represented by $\epsilon_{a}=\mu_{a}=1$, and the non-magnetic metal is represented by $\epsilon_{m}=\epsilon$ and $\mu_{m}=1$. When the real part of the complex dielectric function $\epsilon=\epsilon(\omega, T)$ is much greater than unity, equation S1 can be approximated as:

$R\left( \theta\right)=\frac{\left( n^{2}+k^{2} \right){cos}^{2}\theta-2n\cos\theta+1}{\left( n^{2}+k^{2} \right){cos}^{2}\theta+2n\cos\theta+1}$ (S2)

The pseudo-Brewster angle is defined as the angle where the reflectance is minimized. By differentiating equation S2 with respect to $\theta$, we obtain:

$\cos\theta_{B (M-state)}\sqrt{n^{2}+k^{2}}=$1 (S3)

$\theta_{B (M-state)}=\cos^{-1} (\frac{1}{\sqrt{n^{2}+k^{2}}})$ (S4)

Note S2. Definition of the relative irradiance.

Relative radiance is defined as the ratio of the difference between the radiance of the sample (at temperature 𝑇) and the environment to the difference between the radiance of the control sample (at temperature 𝑇) and the ambient. This is then normalized by the radiance of the control sample at a viewing angle of 0°, as given by the following equation:^[2]^

${L(T)}_{relative}=\frac{{L(T)}_{sample}-{L(T)}_{ambient}}{{L(T)}_{control}-{L(T)}_{ambient}}$ (S5)

${\tilde{L}(T)}_{relative}=\frac{{L\left( T \right)}_{relative}}{Max[{L\left( T \right)}_{control \left( \theta=0^{\circ} \right)}]}$ (S6)

Note S3. Analysis of the IR photographs.

The infrared camera actually collects thermal radiation from the object, the surrounding, and the atmosphere object while measuring the object's radiance, so the total radiant power is given by the following equation:^[3]^

$\Phi_{\det}= \tau_{\mathrm{atm}}\varepsilon\Phi_{\mathrm{object}}^{\mathrm{BB}}\left( T_{\mathrm{object}} \right)+\tau_{\mathrm{atm}}\left( 1-\varepsilon\right)\Phi_{\mathrm{amb}}\left( T_{\mathrm{amb}} \right)+(1-\tau_{\mathrm{atm}})\Phi_{\mathrm{atm}}\left( T_{\mathrm{atm}} \right)$ (S7)

The first term in the equation represents the radiated power collected by the infrared camera from an object with temperature $T_{\mathrm{object}}$. Here, $\tau_{\mathrm{atm}}$ denotes the atmospheric transmittance, $\varepsilon$ is the emissivity of the object, and $\Phi_{\mathrm{object}}^{\mathrm{BB}}\left( T_{\mathrm{object}} \right)$ denotes the thermal radiation emitted by a blackbody with temperature $T_{\mathrm{object}}$. The second term represents the reflected radiant power received by the infrared camera. The reflectivity is calculated from the $\varepsilon$, which is expressed as $1-\varepsilon$. $\tau_{\mathrm{atm}}$ denotes the temperature of the surrounding atmosphere. The last term denotes the radiant power of the atmosphere, and the third term is approximated as 0 due to the short distance (~50 cm) during the actual measurement.

Note S4. Experimental setup for measuring the emissivity spectrum of infrared-transparent BaF_2_ substrate sample.

Considering the infrared transparency of the VO_2_ layer deposited on a barium fluoride (BaF_2_) substrate in the mid-infrared range, emissivity cannot be calculated using the reflectance method as shown in Figure 3D. Therefore, the emissivity of the BaF_2_-based sample was measured using the setup illustrated in Figure S31. During the test, the sample was fixed on a heating stage with a metallic Ag substrate. The thermal radiation emitted by the sample was collected through an external optical path and directed into an FTIR spectrometer (VERTEX 80v, Bruker). A polarizer was included in the optical path to collect thermal radiation signals under different polarization states. A blackbody with a high emissivity (0.95) was used as a reference to calculate the sample's emissivity. The emissivity spectra of the sample after the phase transition were measured at 200 °C, with measurements taken at 10° intervals for angles ranging from 0° to 70° (Figure S18).


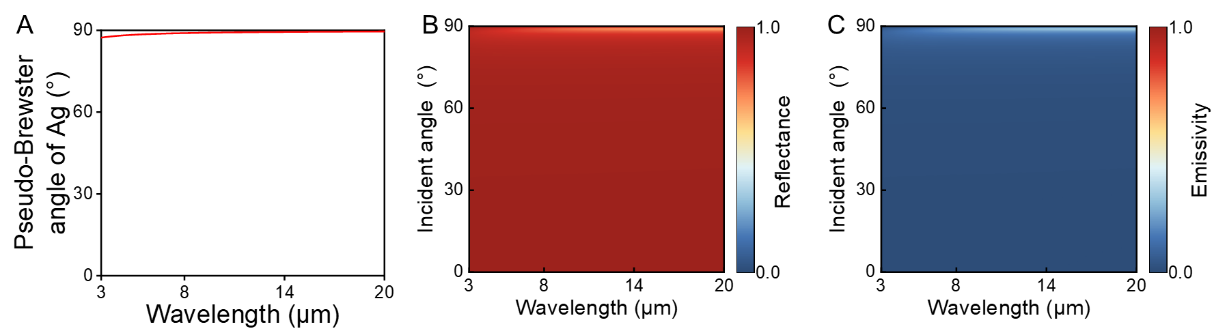


**Figure S1.** (A) Calculated pseudo-Brewster angle of Ag. Calculated angle-dependent reflectance and (B) emissivity (C) spectra of Ag, showing a decrease in reflectance and an increase in absorptive emissivity around 88° due to the pseudo-Brewster effect.


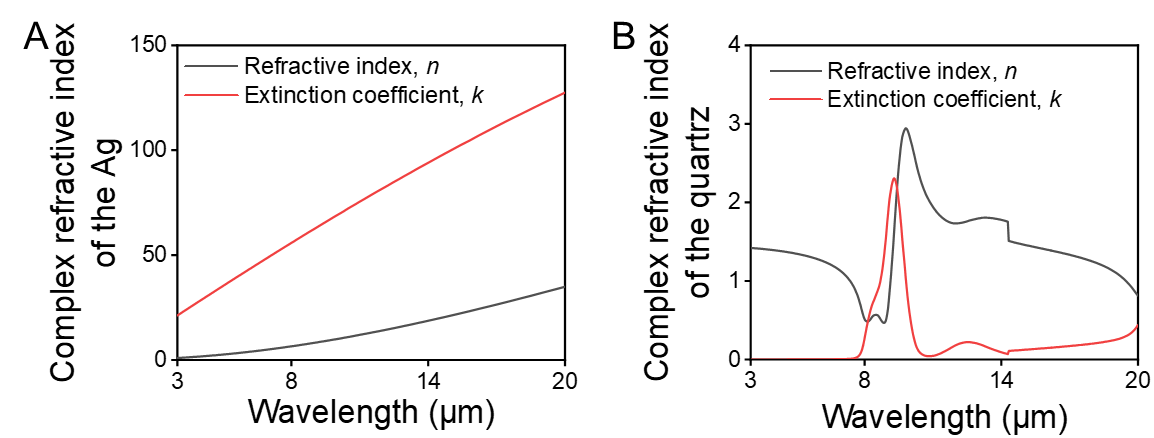


**Figure S2.** Refractive index and extinction coefficient of the Ag (A) and quartz (B).^[4-5]^

**Figure S3.** Calculated emissivity spectra of VO_2_ on Ag layer before and after phase transition using the transfer matrix method (with VO_2_ and substrate thicknesses same as the parameters in Figure 2).


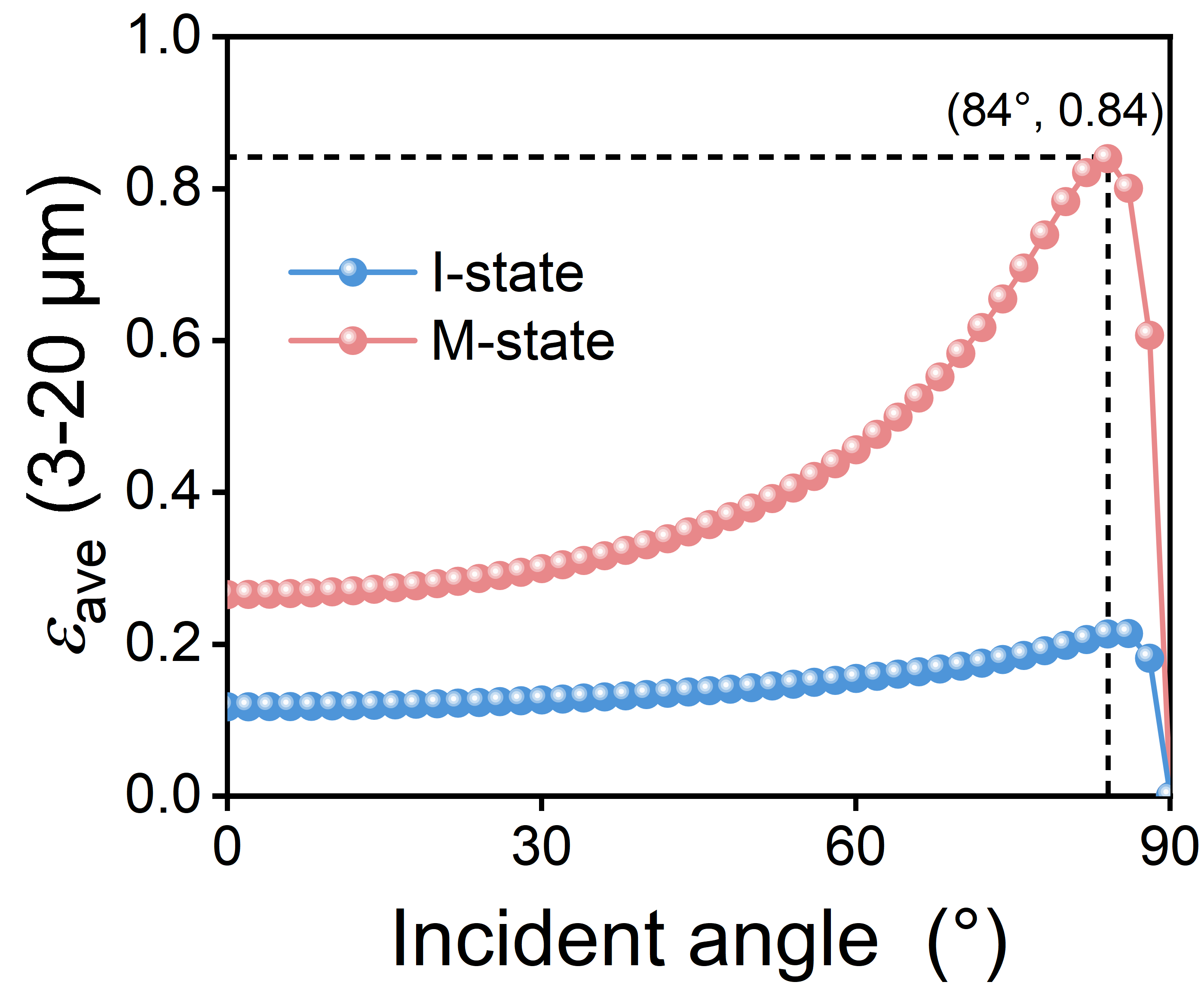


**Figure S4.** Relationship between the average emissivity of the Ag-based UDTTE across the 3–20 µm band and the incident angle.


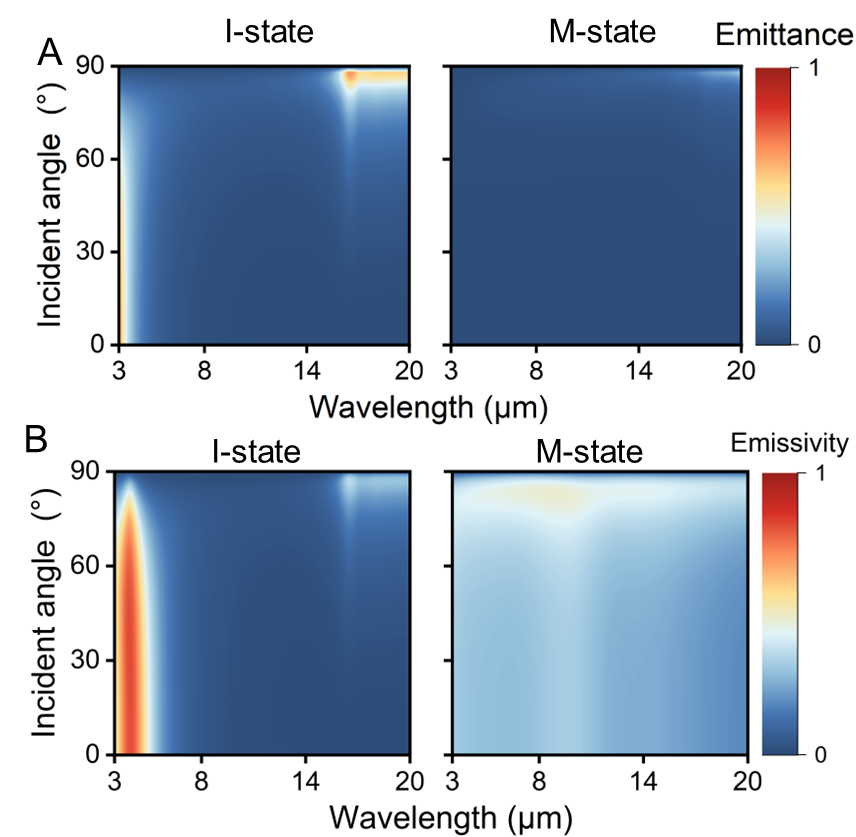


**Figure S5.** Calculated s-polarization (A) and unpolarized (B) angle-dependent emissivity spectra of the UDTTE before (left) and after (right) the phase transition.


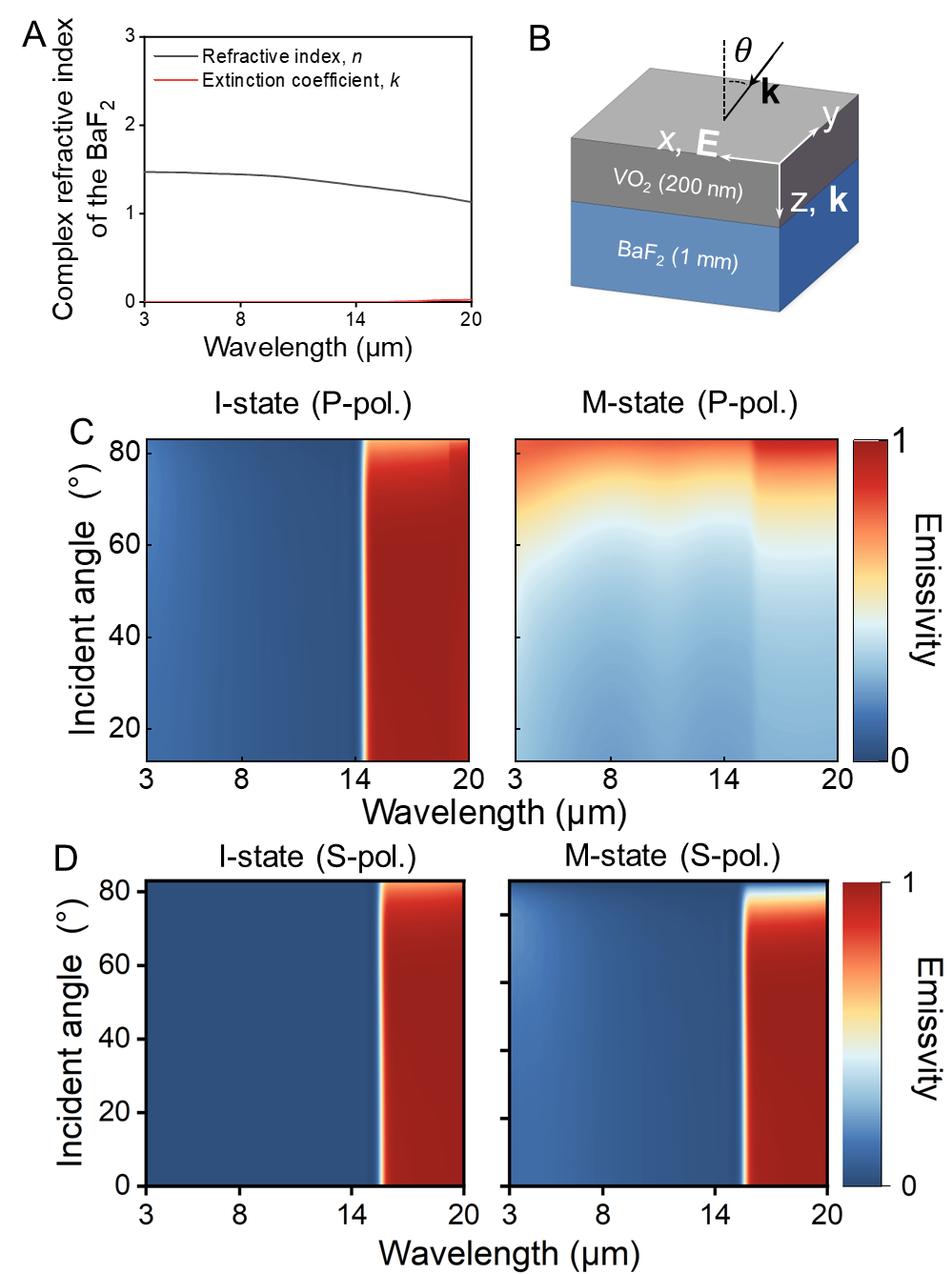


**Figure S6.** (A) Refractive index and extinction coefficient of the Ag. (B) Schematic diagram of the UDTTE based on the IR transparent material BaF_2_. (C) Calculated angle-dependent emissivity spectra of the of the VO_2_ film on a BaF_2_ substrate before (left) and after (right) the phase transition; (D) Calculated angle-dependent emissivity spectra (s-polarization) of the VO2 film on a BaF2 substrate before (A) and after (B) the phase transition.


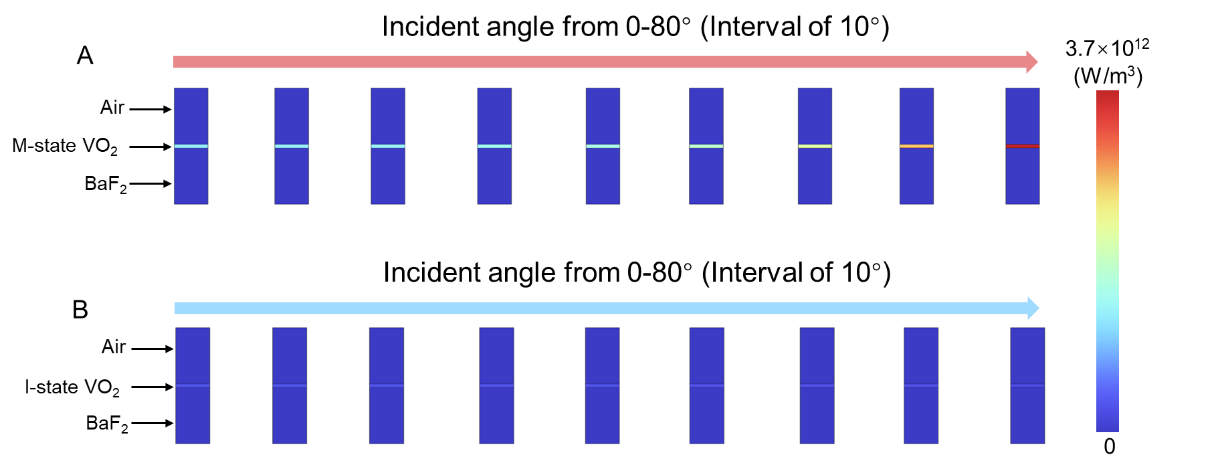


**Figure S7.** Calculated of the power losses distribution of 20 µm electromagnetic waves incident on M-state (B) and I-state (B) VO_2_ (200 nm)/BaF_2_ (1 mm).


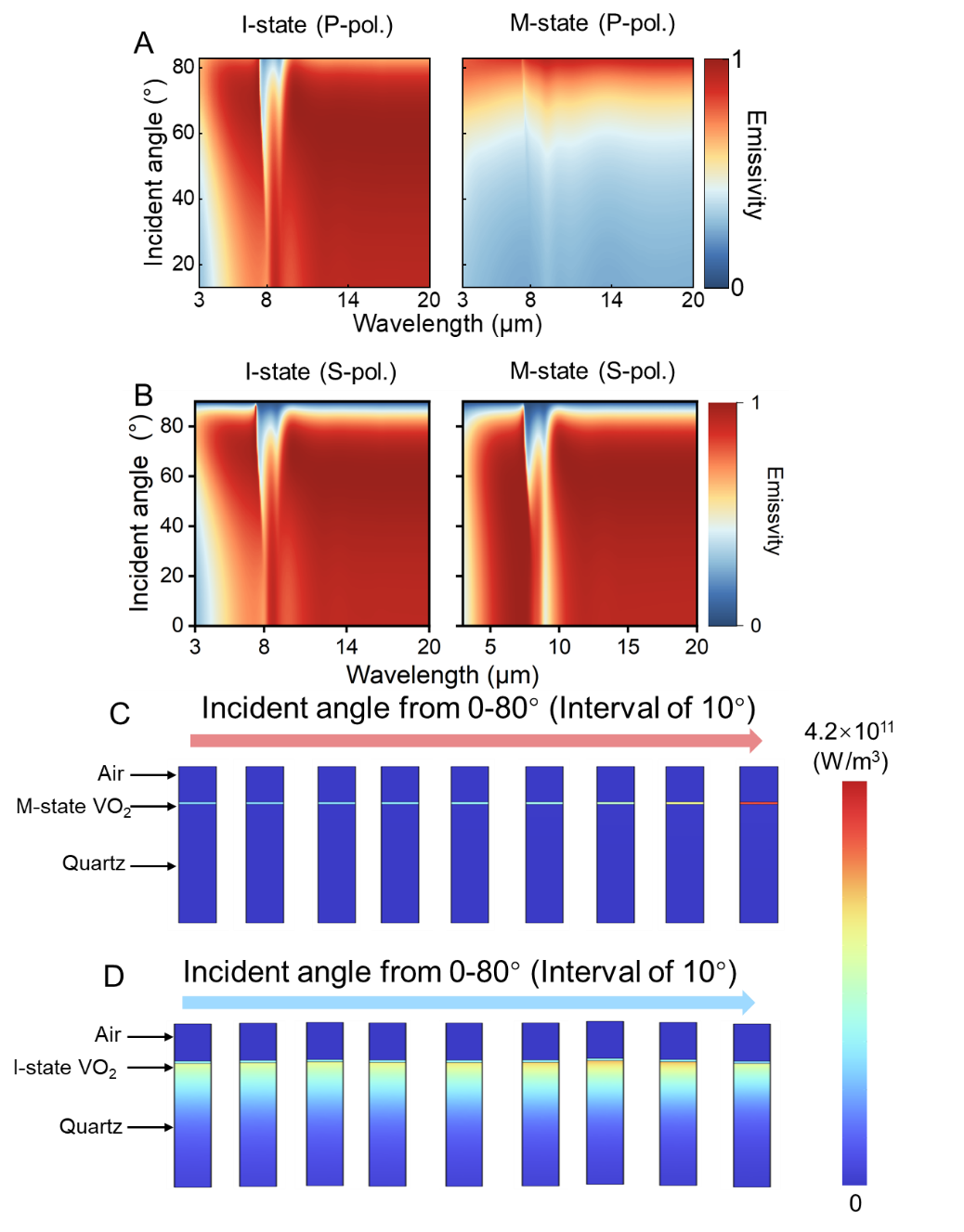


**Figure S8.** (A) Calculated angle-dependent emissivity spectra (P-polarization) of the VO_2_ film based on a quartz substrate before (left) and after (right) the phase transition. (B) Calculated angle-dependent emissivity spectra (s-polarization) of the VO_2_ film based on a quartz substrate before and after the phase transition. Calculated of resistive power losses distribution of 20 µm electromagnetic waves incident on M-state (C) and I-state (D) VO2 (300 nm)/quartz (1 mm).

**Figure S9.** Schematic diagram of the process for preparing UDTTE using magnetron sputtering.


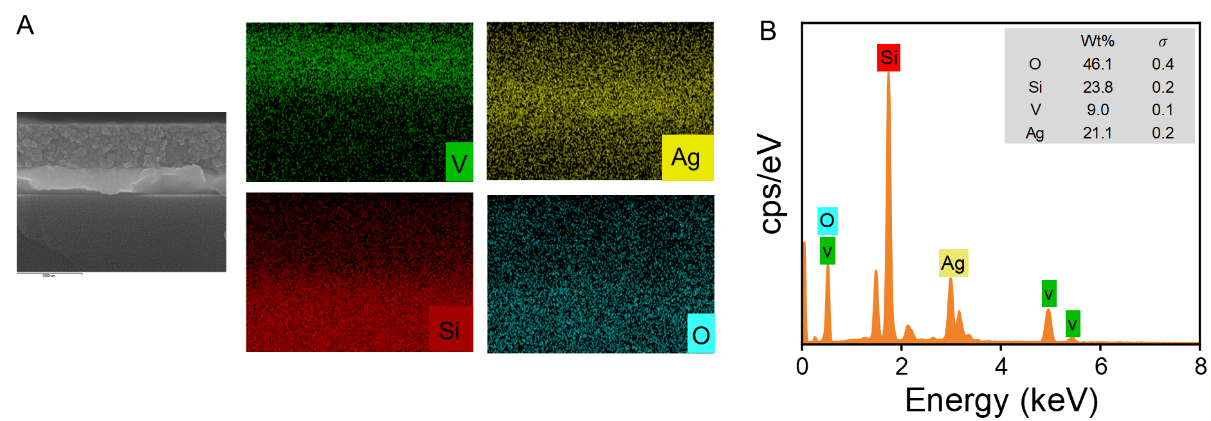


**Figure S10.** Surface-scanning (A) and point-scanning (B) energy dispersive spectroscopy (EDS) spectroscopy of the UDTTE.


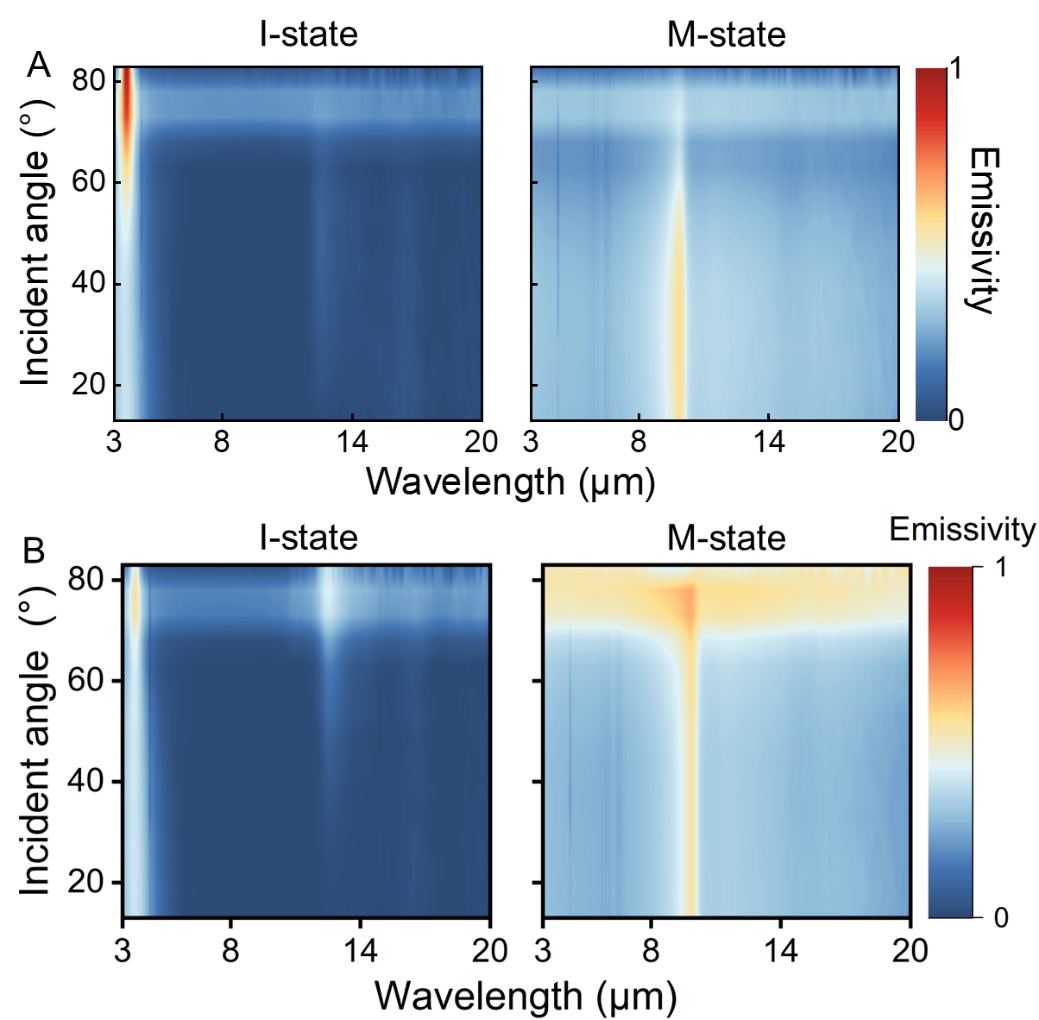


**Figure S11.** Measured angle-dependent s-polarization (A) and unpolarized (B) emissivity spectra of the UDTTE before (left) and after (right) the phase transition.


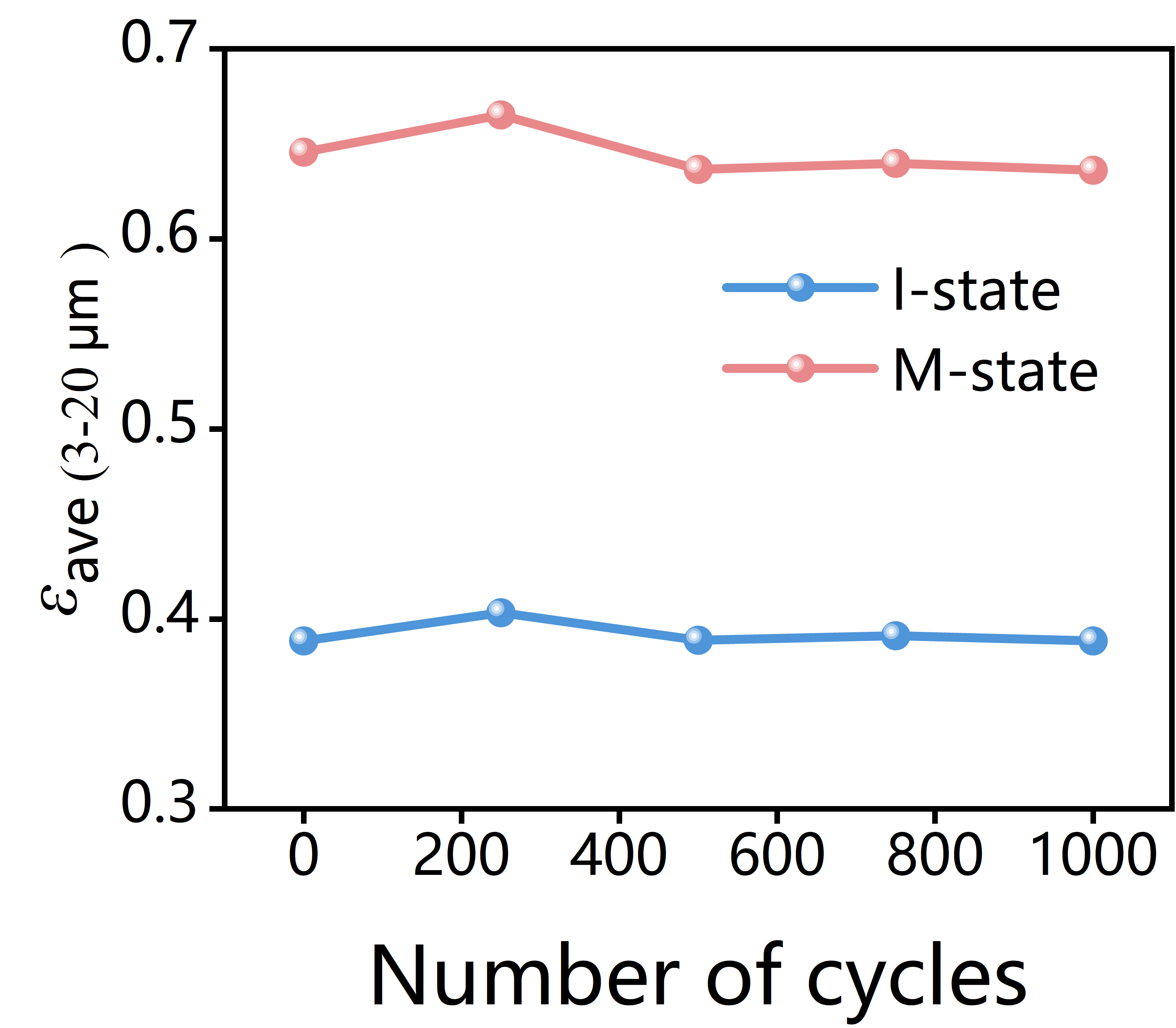


**Figure S12.** (A) Average emissivity of the VO₂ film (on a quartz substrate) before and after the phase transition, measured before and after high-low temperature cycling (30-80 ℃).


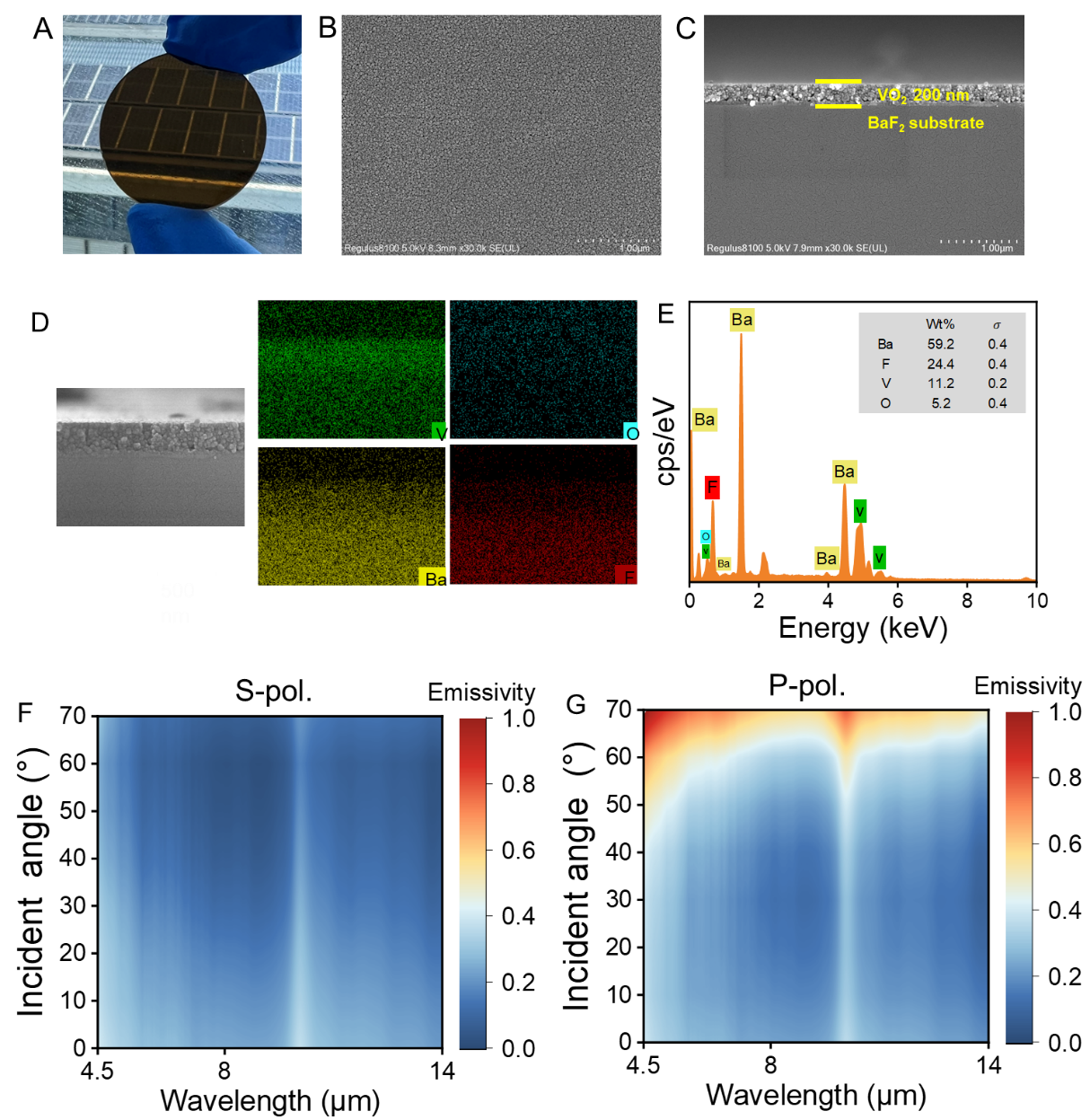


**Figure S13.** Optical photograph (A), Surface (B) and cross-sectional (C) SEM images of VO_2_ (200 nm)/BaF_2_ (1 mm) structure captured using a scanning electron microscope (SEM). Surface-scanning EDS spectrum (D) and point-scanning EDS spectrum (E) of the VO_2_ (200 nm)/BaF_2_ (1 mm) structure. Measured angle-dependent emissivity spectra of VO_2_ (200 nm)/BaF_2_ (1 mm) structure at high temperature (200 °C) under S-polarization (F) and P-polarization (G) measured using the blackbody comparison method (for more details, see Note S4).


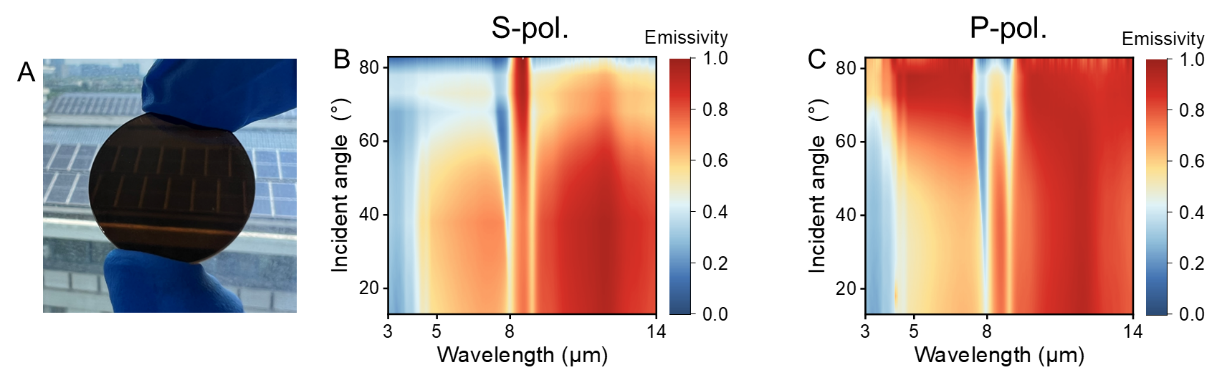


**Figure S14** (A) Optical photograph of the VO_2_ (300 nm) /quartz (1 mm) structure. Measured angle-dependent emissivity spectra of the VO_2_ (300 nm) /quartz structure (1 mm) at low temperature (26 °C) under S-polarization (B) and P-polarization (C).


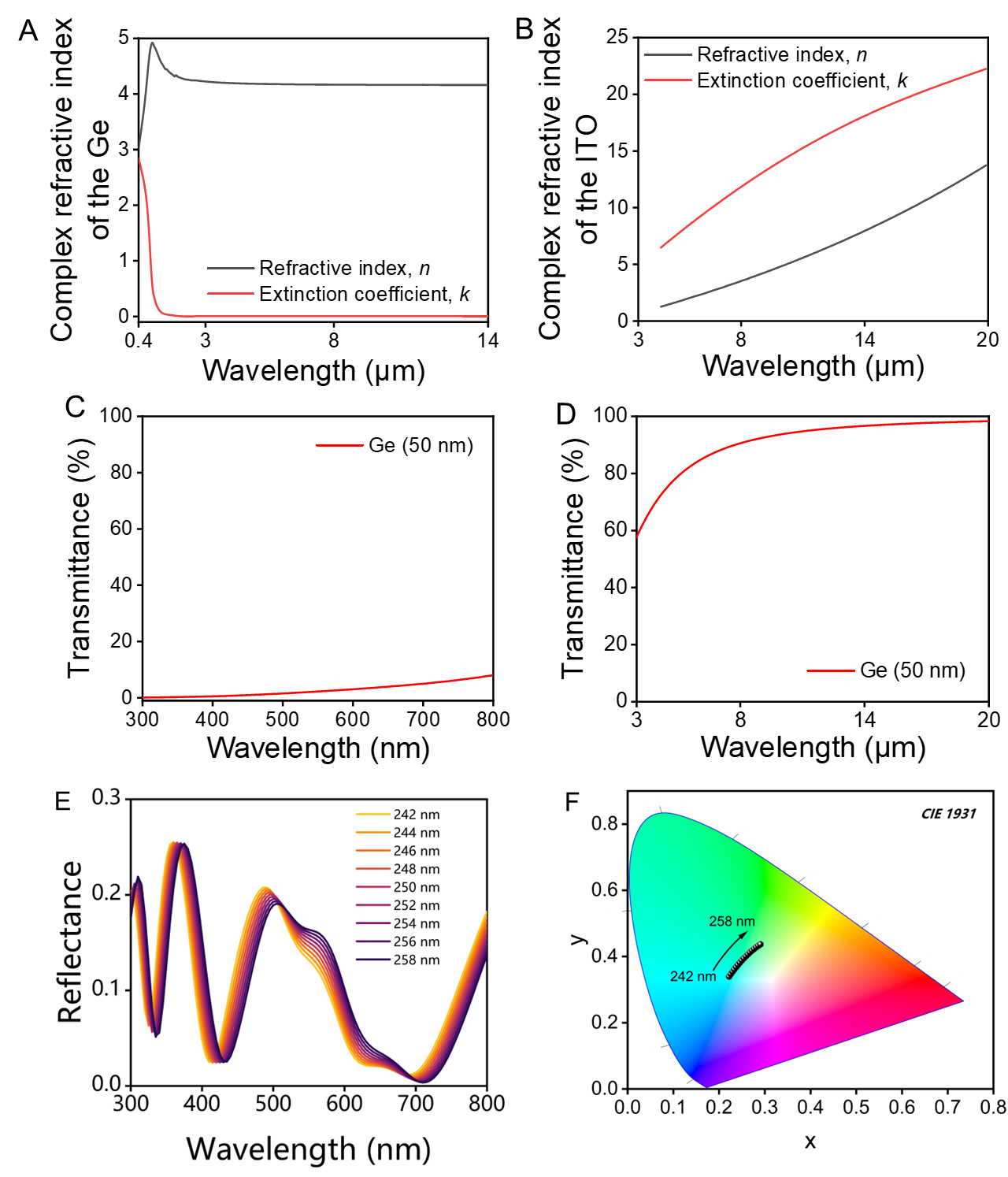


**Figure S15.** Refractive index and extinction coefficient of the Ge (A) and ITO (B).^[6-7]^ Calculated transmittance of the 50-nm-thick Ge in the visible (C) and mid-infrared (D) bands. (E) Calculated visible light reflectance of UDTTE when the thickness of the ITO layer varies between 242 nm and 258 nm. (F) Distribution of visible light color coordinates corresponding to ITO thickness variations between 242 nm and 258 nm.


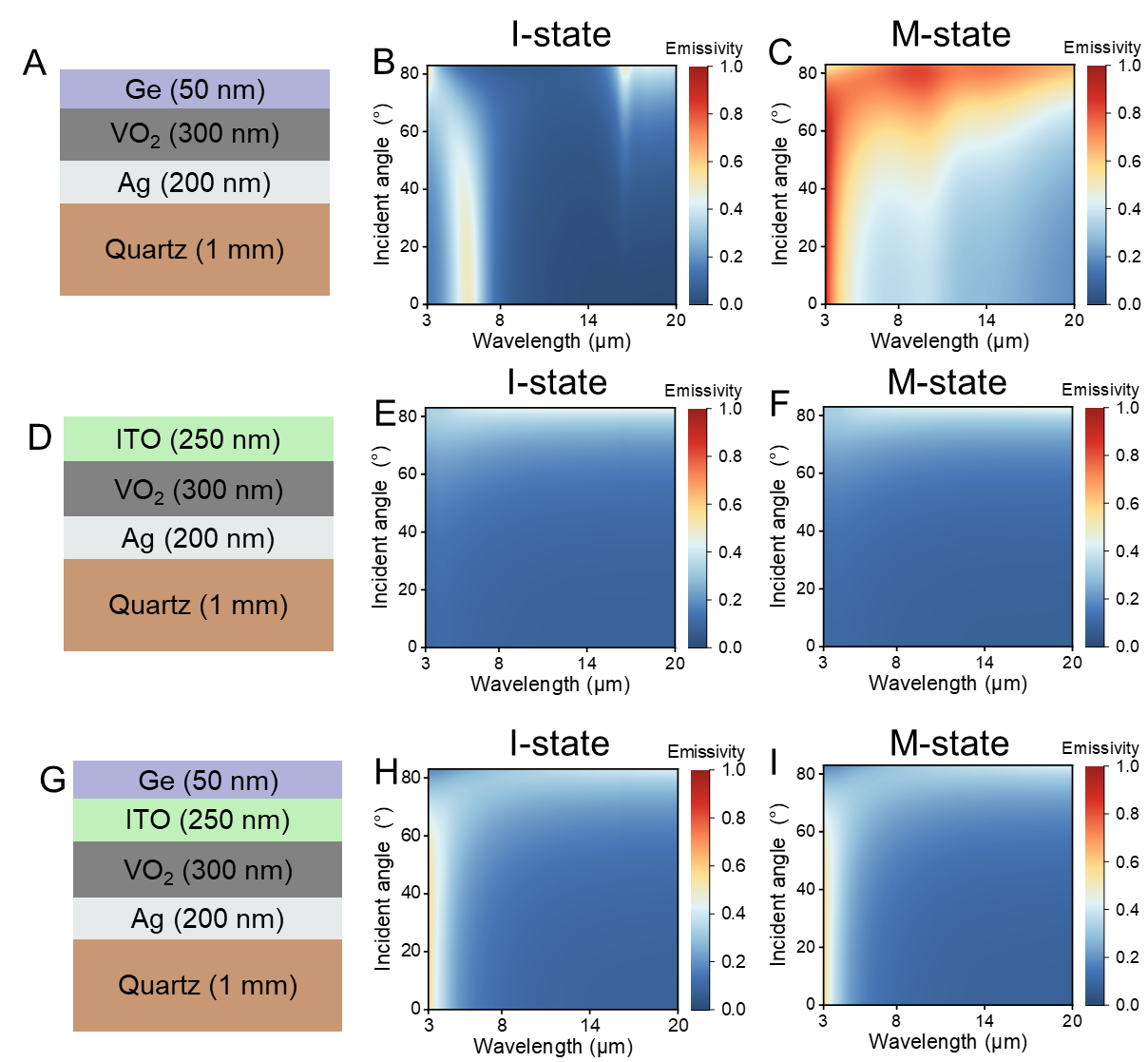


**Figure S16.** (A) Schematic diagram of the UDTTE structure with an added 50 nm Ge layer. Calculated angle-dependent emissivity spectrum of the UDTTE with the 50 nm Ge layer before (B) and after (C) the phase transition. (D) Schematic diagram of the UDTTE structure with an added 250 nm ITO layer. Calculated angle-dependent emissivity spectrum of the UDTTE with the 250 nm ITO layer before (E) and after (F) the phase transition. (G) Schematic diagram of the UDTTE structure with an added 50 nm Ge layer and 250 nm ITO layer. Calculated angle-dependent emissivity spectrum of the UDTTE with the 250 nm ITO layer before (H) and after (I) the phase transition.


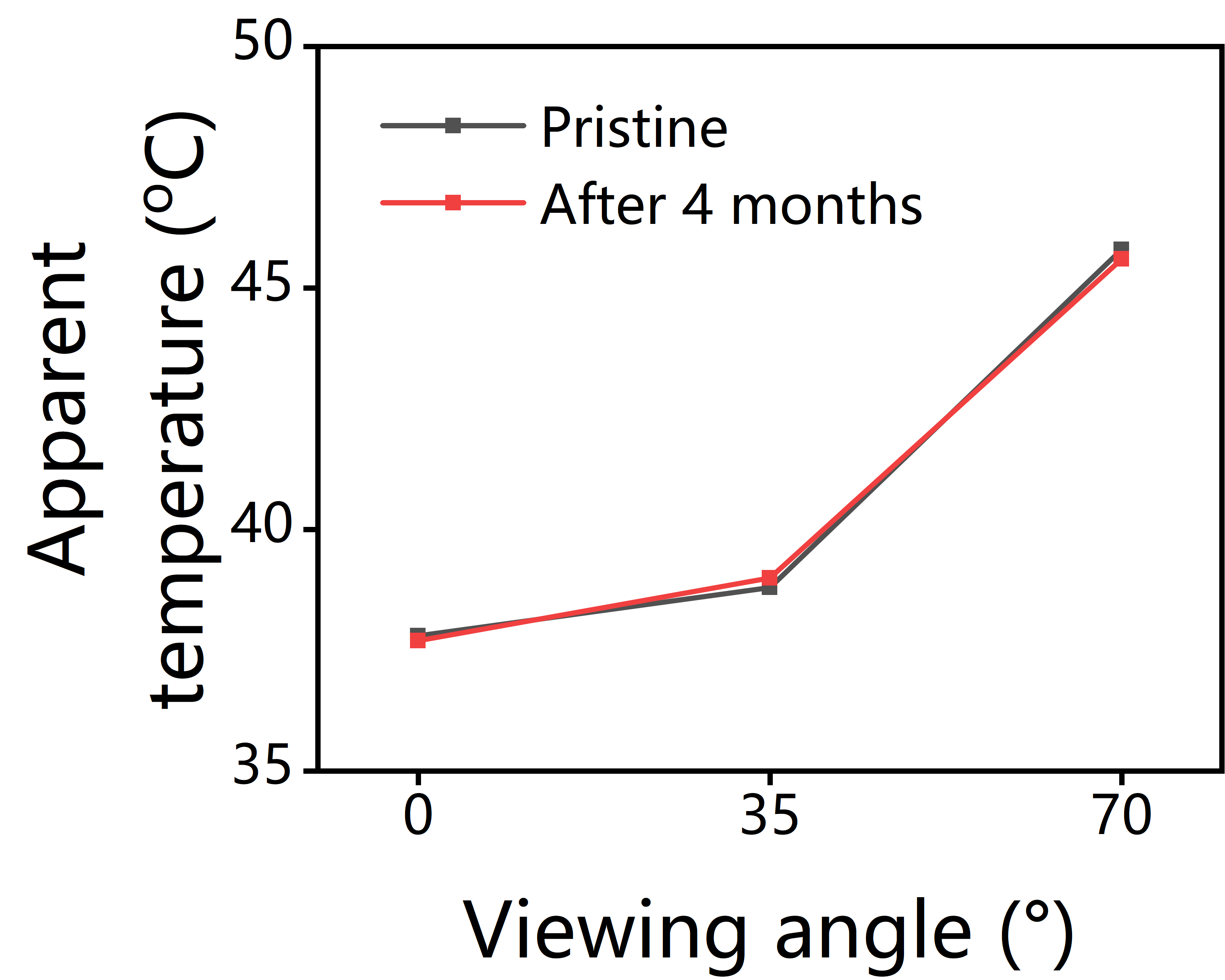


**Figure S17.** Relationship between apparent temperature and viewing angle for the information encryption UDTTE under p-polarization before and after four months of air exposure.


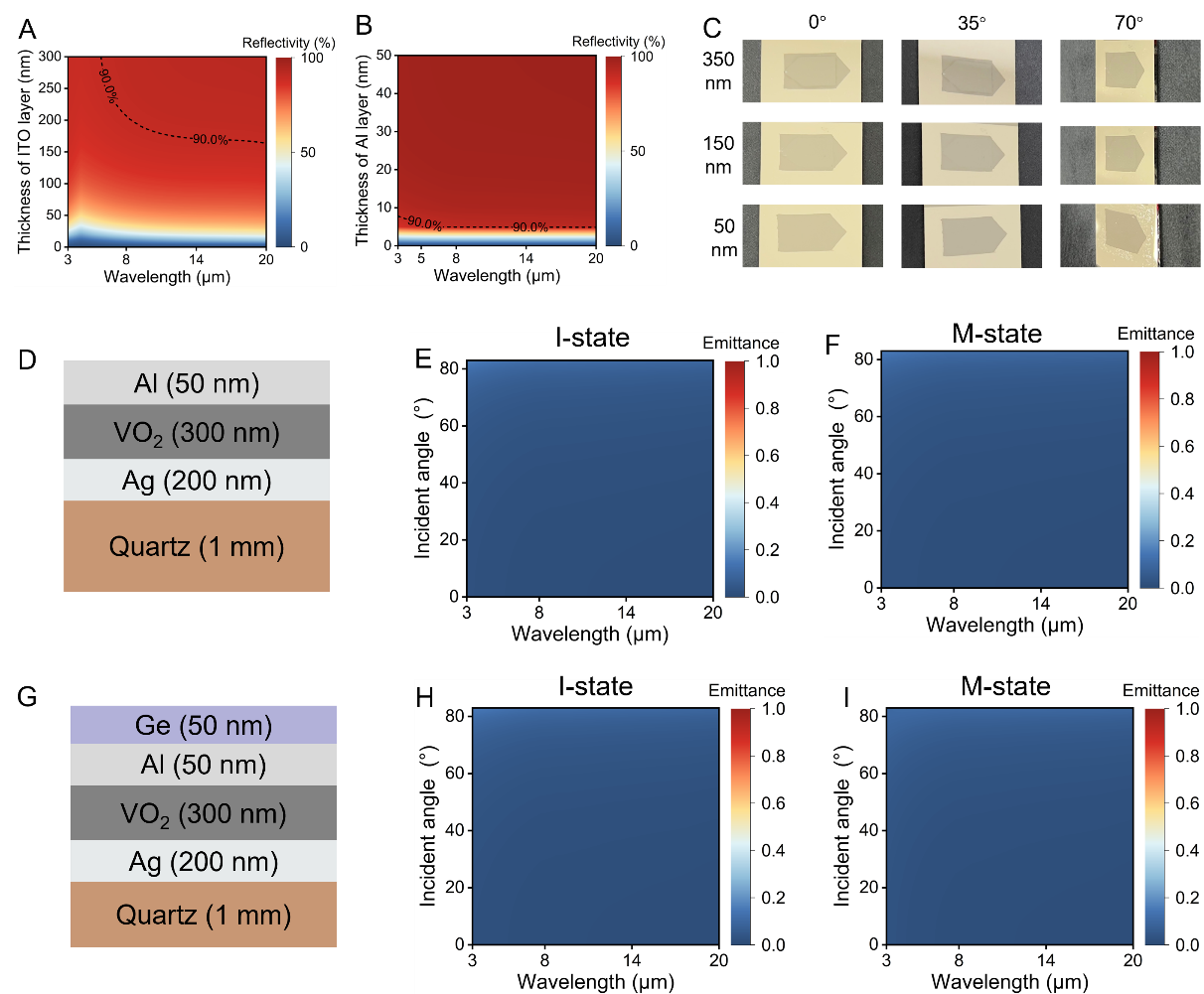


**Figure S18.** (A) Calculated relationship between the thickness of the ITO layer and its emissivity. (B) Calculated relationship between the thickness of the Al layer and its emissivity. (C) Optical photographs of Al layers with thicknesses of 50, 150, and 350 nm used as low-emissivity background layers in information encryption demonstrations. (D) Schematic diagram of the UDTTE with an added 50 nm Al layer. Calculated angle-dependent emissivity spectra of the UDTTE with the 50 nm Al layer before (E) and after (F) the phase transition. (G) Schematic diagram of the UDTTE with an added 50 nm Al and 50 nm Ge layer. Calculated angle-dependent emissivity spectra of the UDTTE with the 50 nm Al and 50 nm Ge layers before (H) and after (I) the phase transition.


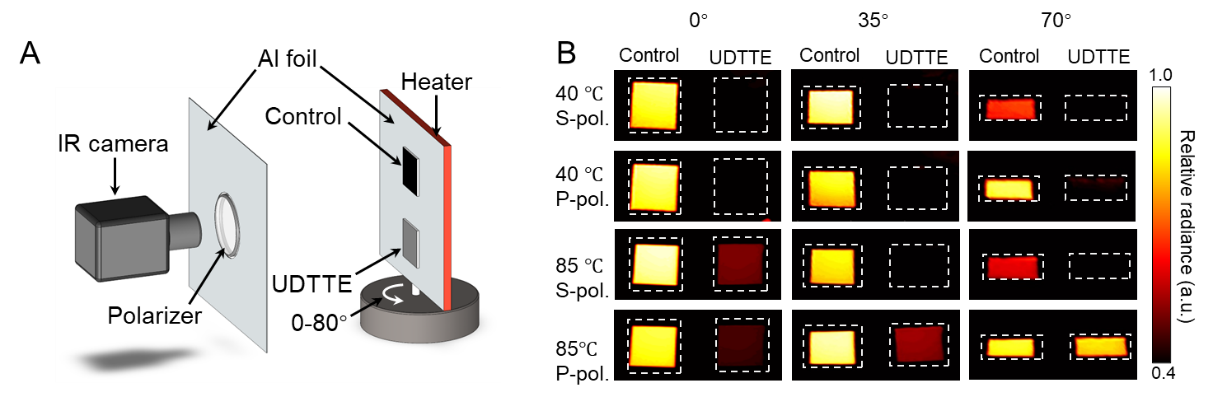


**Figure S19.** (A) Schematic diagram of the setup demonstrating multi-level IR information encryption using the UDTTE with an IR camera. (B) IR images of the control sample and UDTTE at different temperatures, polarizations, and angles.


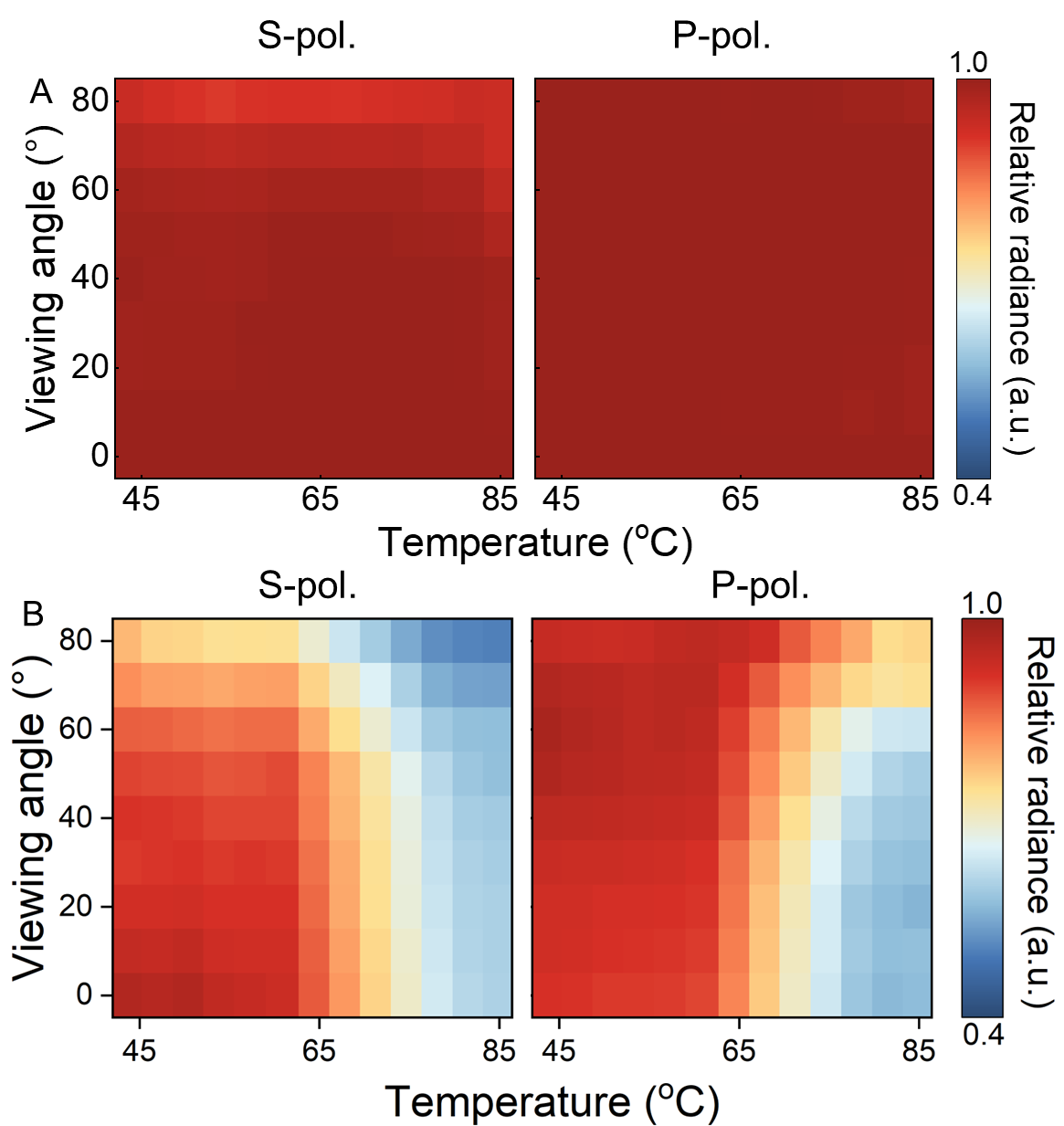


**Figure S20. (A)** Relative radiance of the control sample at different polarizations, temperatures, and viewing angles. (B) Relative radiance of the VO_2_ (300 nm)/quartz (1 mm) structure at different polarizations, temperatures, and viewing angles.


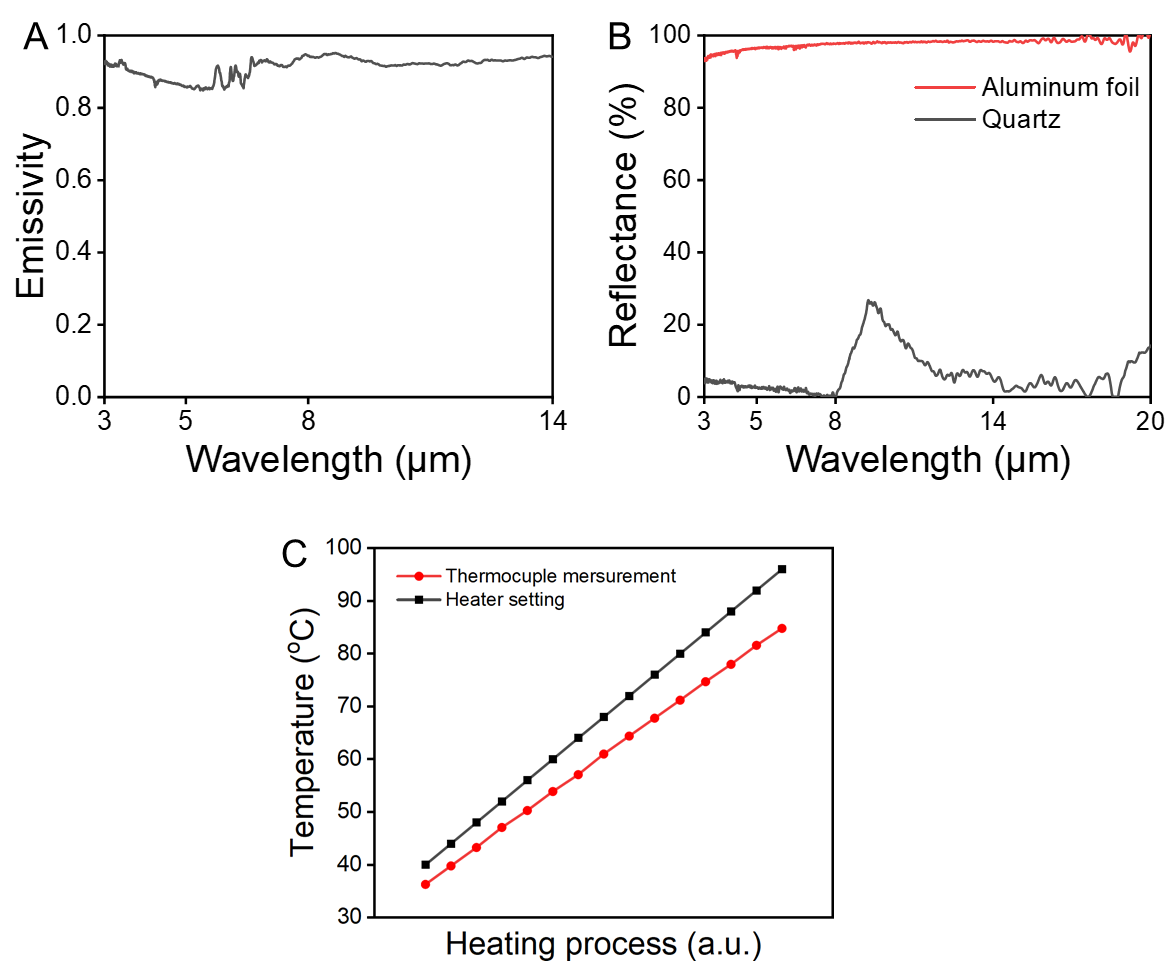


**Figure S21.** Measured reflectance spectrum of the aluminum foil and quartz. (B) Measured emissivity spectrum of the high-emissivity paint. (C) Comparison between the set temperature of the heater and the actual sample temperature measured by the K-type thermocouple during the multi-level infrared information encryption experiment.


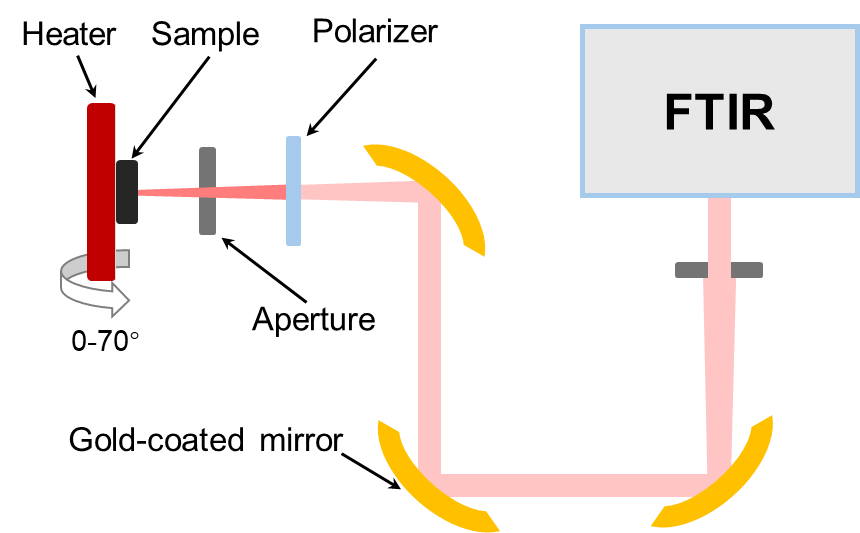


**Figure S22.** The schematic diagram of the measured setup for measuring the angle-dependent emissivity spectra of the BaF2-based sample.

**Table R1.** Performance Comparison of the Ag-based, BaF₂-based, and Quartz-based UDTTE.

|  | Ag-based | BaF_2_ -based | Quartz-based |
| --- | --- | --- | --- |
| Directional emission | Yes | Yes | Yes |
| Tunability | Yes | Yes | Yes |
| Switching Mechanism | Omnidirectional low-𝜀/Directional high-𝜀 | Omnidirectional low-𝜀/Directional high-𝜀 | Omnidirectional high-𝜀/Directional high-𝜀 |
| Spectral Range | Ultrabroadband (3-20 µm) | Broadband (3-14 µm) | Ultrabroadband  (3-20 µm) |
| Preparation costs | Low | High | Low |

References

[1] B. Hüttner, *J. Appl. Phys.* **1995**, 78, 4799.

[2] S. An, W. Shang, M. Jiang, Y. Luo, B. Fu, C. Song, P. Tao, T. Deng, Proc. *Natl. Acad. Sci. U.S.A.* **2021**, 118, e2021077118.

[3] Infrared Thermal Imaging: Fundamentals, Research and Applications, *European Journal of Physics,* **2011** 32 1431.

[4] J. Kischkat, S. Peters, B. Gruska, M. Semtsiv, M. Chashnikova, M. Klinkmüller, O. Fedosenko, S. Machulik, A. Aleksandrova, G. Monastyrskyi, Y. Flores, W. Ted Masselink, *Appl. Opt.* **2012**, 51, 6789.

[5] H. U. Yang, J. D'Archangel, M. L. Sundheimer, E. Tucker, G. D. Boreman, M. B. Raschke, *Phys. Rev. B* **2015**, 91, 235137.

[6] T. Amotchkina, M. Trubetskov, D. Hahner, V. Pervak, Appl. Opt. **2020**, 59, A40.

[7] M. Bae, D. H. Kim, S.-K. Kim, Y. M. Song, *Nanophotonics* **2024**, 13, 749.
